# Thermo-Magnetic Induction of Pro-Inflammatory Microglia: A Lipid-Based Nanovector Strategy for Glioblastoma Immunotherapy

**DOI:** 10.1021/acsami.5c18518

**Published:** 2025-11-05

**Authors:** Maria Cristina Ceccarelli, Giuliana Paravizzini, Attilio Marino, Giulia Gigante, Alessio Carmignani, Federico Catalano, Mirko Prato, Giammarino Pugliese, Pietro Fiaschi, Matteo Battaglini, Gianni Ciofani

**Affiliations:** † 121451 Istituto Italiano di Tecnologia, Smart Bio-Interfaces, Viale Rinaldo Piaggio 34, 56025 Pontedera, Italy; ‡ Scuola Superiore Sant’Anna, The BioRobotics Institute, Viale Rinaldo Piaggio 34, 56025 Pontedera, Italy; § Department of Mechanical and Aerospace Engineering, 19032 Politecnico di Torino, Corso Duca degli Abruzzi 24, 10129 Torino, Italy; ∥ Istituto Italiano di Tecnologia, Electron Microscopy Facility, Via Morego 30, 16163 Genova, Italy; ⊥ Istituto Italiano di Tecnologia, Materials Characterization Facility, Via Morego 30, 16163 Genova, Italy; # Istituto Italiano di Tecnologia, Chemistry Facility, Via Morego 30, 16163 Genova, Italy; ∇ Department of Neurosurgery, 9246 IRCCS Ospedale Policlinico San Martino, Largo Rossana Benzi 10, 16132 Genova, Italy; ○ Department of Neuroscience, Rehabilitation, Ophthalmology, Genetics, Maternal and Child Health (DiNOGMI), University of Genova, Largo Paolo Daneo 3, 16132 Genova, Italy

**Keywords:** microglia modulation, lipid-magnetic nanoparticles, hyperthermia, glioblastoma, immunotherapy

## Abstract

Microglia, the main immune cells in the central nervous system (CNS), maintain physiological homeostasis and react to pathological changes. Besides their neuroprotective function, they play a crucial role in brain tumor microenvironments such as glioblastoma (GBM), by composing up 40% of the tumor mass. Glioma-associated microglia exhibit a dynamic activation state characterized mainly by an immunosuppressive (M2-like) response, with a lesser contribution of pro-inflammatory (M1-like) response. Modulating microglial into M1-like phenotype offers antitumor response and a promising immunotherapy strategy against GBM. Nanoparticles can induce microglial polarization, also modulating pro-inflammatory responses for tumor suppression. Magnetically responsive nanoparticles are promising nanotransducers due to their remote-control capabilities via external magnetic fields, enabling precise therapeutic interventions. This study proposes a novel strategy that exploits lipid-based magnetic nanovectors (LMNVs) composed of a lipid matrix doped with iron oxide nanoparticles to induce M1-like microglial response through magneto-thermal conversion. Results demonstrated that LMNVs exhibit excellent biocompatibility and efficient internalization within human microglia (HMC3 cells). Upon alternating magnetic field (AMF) stimulation, LMNVs triggered a sustained increase in intracellular Ca^2^^+^ levels, leading to the polarization of microglia toward a pro-inflammatory M1-like phenotype. This activation was confirmed by the upregulation of key inflammatory markers (CD40, CD86) and cytokine release (IL-6, IL-8, and TNF-α), mirroring the effects of IFN-γ stimulation. These findings were further corroborated by comparative transcriptomic analysis. Notably, conditioned medium from LMNVs + AMF-stimulated microglia significantly impaired the viability and proliferation of both immortalized and patient-derived GBM cells, demonstrating a potent antitumor response. The tumor cell death was associated with immunogenic cell death (ICD), as indicated by the translocation of the damage-associated molecular patterns, in particular high mobility group box 1 (HMGB1) and calreticulin (CRT). Overall, these results highlight the potential of LMNVs as a remotely activatable nanoplatform capable of reprogramming microglia and to promote antitumor immunity in GBM.

## Graphical abstract

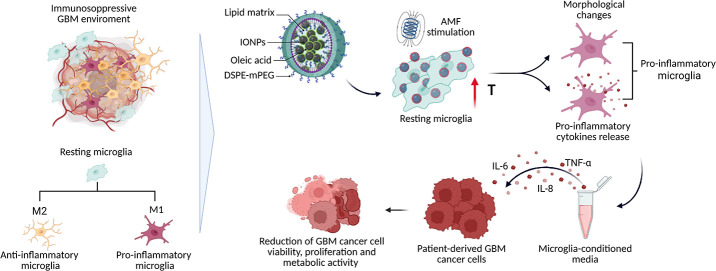

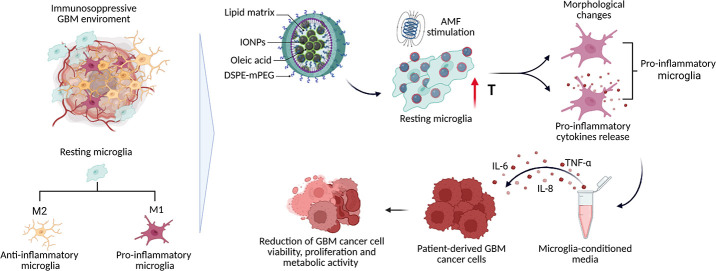

## 1 Introduction

Microglia represent the main immune cells of the central nervous system (CNS), playing essential roles in supporting neuronal health as well as maintaining physiological homeostasis. They monitor the CNS for potential pathological events, and to contrast these changes, microglia act with protective mechanisms. These cells can exhibit protective functions by phagocytosing and clearing pathological protein aggregates; however, the excessive uptake can compromise their phagocytic ability, leading to neuroinflammation and subsequent neurodegeneration.^1^ Due to their immune function, microglia are found in nearly all CNS disorders, including malignant brain tumors.

Glioma-associated microglia and macrophages (GAMs) represent the largest proportion of immune infiltrating cells in gliomas, comprising up to 40% of the tumor mass.^2^ Their interactions with glioma cells drive a complex and dynamic activation state characterized mainly by an immunosuppressive response (M2-like phenotype), with a lesser contribution of pro-inflammatory response (M1-like phenotype).^1^ While microglia have the potential to induce an antitumor response, glioma cells counteract this effect by releasing immunosuppressive factors, effectively inactivating their defense mechanisms. At the same time, microglia secrete a range of immunomodulatory cytokines that support tumor growth.^3^ However, this traditional M1/M2 classification of microglial activation oversimplifies the various activation states of these cells, and does not adequately capture their functional plasticity within the glioma microenvironment.^3,4^ Recent research indicates that microglia exist along a continuum of activation states rather than as distinct. Their response to signals derived from gliomas results in a range of transcriptional and functional changes, which are influenced by both tumor-secreted and local microenvironmental factors.^4^ This complex behavior highlights the necessity for a new nomenclature that better represents the heterogeneous and context-dependent roles of microglia in GBM. Nevertheless, classifying microglia activation according to a traditional simplified model can help to clarify their role in CNS disorders and the therapeutic potential of immunomodulation.

Modulating microglial activity, especially promoting a pro-inflammatory phenotype that acts as an antitumor factor, has emerged as a promising therapeutic strategy against GBM. In particular, nanomaterials have been identified as promising tools for manipulating microglial responses, offering the potential to specifically target these cells and induce in situ immunomodulation.^5^ Recent studies have explored various nanostructures as immunomodulators capable of influencing microglial polarization, aiming to stimulate a pro-inflammatory response against brain cancers.^6^ These strategies can be categorized into three main approaches: active modulation, passive modulation, and remotely activated modulation. An example of active modulation is provided by Wang et al., showing how doxorubicin-loaded zinc oxide nanoparticles (ZnO-DOX) could induce M1-like polarization in macrophages.^7^ The treatment led to increased expression of pro-inflammatory markers and cytokines, contributing to an antitumor response. The study suggests that ZnO-DOX nanoparticles can modulate the tumor microenvironment by stimulating macrophages to upregulate the expression of pro-inflammatory markers such as CD80 and CD86.^7^ Similar results in terms of microglia polarization were observed with silica nanoparticles (SiNPs) and iron oxide nanoparticles (IONPs). These nanoparticles (NPs), due to their intrinsic physicochemical properties and surface functionalization, can directly influence microglial behavior, promoting a pro-inflammatory phenotype without the need of external activation or drug loading.^6^ Furthermore, positively charged IONPs enhance interactions with microglia, leading to pro-inflammatory responses that are characteristic of M1-like polarization.^8^

Building on these findings, additional nanosystems have emerged to further expand the immunomodulatory toolkit. In addition to previously reported NPs such as ZnO, SiNPs, and IONPs, polymeric nanocarriers based on poly­(β-amino ester) (PbAE) have been engineered to codeliver mRNA encoding interferon regulatory factor 5 (IRF5) and IKKβ, two critical transcription factors for M1-like polarization. In preclinical GBM models, this approach significantly increased the expression of M1-associated cytokines, suppressed tumor growth, and extended survival.^9^ Another innovative system involves DNA-grafted polycaprolactone brush (DNA-*g*-PCL) nanostructures cross-linked with microRNA-155, coated with red blood cell membranes for immune evasion and functionalized with M2-targeting peptides (M2pep). These NPs selectively accumulate in M2-like microglia/macrophages and successfully reprogram them toward an M1-like phenotype, resulting in reduced tumor burden in glioma-bearing mice.^10^

Recent advancements have led to the development of NPs capable of remotely modulating microglial activity toward the M1-like pro-inflammatory phenotype upon exposure to external stimuli, such as ultrasound or magnetic fields. This strategy can represent a cutting-edge strategy to influence microglial polarization and enhance antitumor responses.

A promising example of this approach is illustrated by a previous work from our group^11^ where piezoelectric nanoparticles (PNPs) coated with extracts from GBM cell membranes were designed to respond to external ultrasound stimulation. This mechanism works by harnessing the electrical charge generated by PNPs under ultrasound exposure, which in turn activates microglial cells and promotes the release of pro-inflammatory cytokines, reducing the GBM cell viability and proliferation. Although ultrasound-activated piezoelectric NPs have significant potential, the technique faces several limitations. One of the most significant challenges is the penetration depth of ultrasound waves. Ultrasound, although noninvasive, often struggles to effectively reach deeper brain tissues, especially when tumors are located deeper within the brain or surrounded by dense tissue structures such as bone or fluid.^12^ This reduced penetration can limit the therapeutic effect of ultrasound-based treatments, making it less effective for treating deep-seated tumors.^12^ Moreover, the precision and control of ultrasound stimulation are crucial in ensuring targeted activation of the nanoparticles. Variations in tissue composition or skull structure across patients can alter how ultrasound waves propagate, leading to inconsistent or suboptimal therapeutic outcomes.^13^ Given these limitations, exploring alternative external stimuli, such as magnetic fields, may offer a more effective and reliable approach for remotely activated modulation. Magnetic nanoparticles (MNPs), in particular, are responsive to external magnetic fields, enabling precise targeting and activation even in deep brain regions. Unlike ultrasound, magnetic fields can penetrate tissues more uniformly and provide greater control over particle movement and activation, offering a promising avenue for improving the efficacy of remotely activated treatments in brain cancer.^14^

In this work, we propose a novel approach to induce an antitumor microglial response using MNPs; the present study aims to combine alternating magnetic field (AMF) stimulation with IONPs embedded in a lipid matrix to create lipid-based magnetic nanovectors (LMNVs). The designed LMNVs demonstrated excellent biocompatibility and efficient cell internalization in human microglia cells (HMC3). The magneto-thermal conversion exhibited by LMNVs in combination with chronic AMF stimulation induced marker expression, secretion of inflammatory cytokines, and upregulation of relevant gene transcription in accordance with a M1-like phenotype. In particular, a significant reduction in cell viability, proliferation, and metabolic activity was observed in U87-MG and patient-derived GBM cells following treatment with microglia-conditioned media from LMNV-treated samples in combination with chronic AMF stimulation. To further support the induction of an immunogenic antitumor response, we evaluated the expression of immunological cell death (ICD) markers: our results showed a clear cytoplasmic translocation of high mobility group box 1 (HMGB1) from the nucleus and a strong surface positivity for calreticulin (CRT) upon LMNVs + AMF treatment. Obtained results were corroborated by a comparative transcriptomic analysis. The data revealed upregulation of genes associated with homeostatic regulation in AMF- and LMNVs-treated cells, while condition-specific mechanisms driving pro-inflammatory activation and immunogenic cell death, along with morphological changes, were observed in LMNVs + AMF and IFN-γ groups.

Overall, the proposed stimulation approach enhances antitumor immune responses against both immortalized and patient-derived GBM cells, highlighting the potential of these nanoparticles as a promising immunomodulatory platform.

## 2 Materials and Methods

### 2.1 Nanoparticle Synthesis and Characterization

The synthesis of IONPs was performed following an optimized procedure described in a previous work.^15^ The iron oleate precursor was prepared by reacting 1.8 g of iron­(III) chloride (FeCl_3_, Sigma-Aldrich) with 6.3 g of sodium oleate (TCI) in a biphasic system consisting of 10 mL of Milli-Q water (Millipore), 13.35 mL of ethanol, and 23.35 mL of hexane, all under a nitrogen flow. This mixture was heated to 70 °C and refluxed for 2 h. After the reaction, the phases were allowed to separate, and the solvents were removed using a rotary evaporator, followed by vacuum drying at 50 °C overnight. For the synthesis of IONPs, 6 g of the iron oleate precursor was mixed with 25.4 g of eicosane (TCI) and 3.66 mL of oleic acid (Sigma-Aldrich). The thermal decomposition of this mixture was conducted under an argon atmosphere, where the temperature was gradually increased to 350 °C at a rate of 2 °C/min and maintained for 30 min. To enhance the Fe^3^^+^ content and facilitate the crystallization into the magnetite phase, an oxidation step was implemented. This involved adding 1.2 g of trimethylamine *N*-oxide (TCI) to the freshly synthesized IONPs, followed by annealing under argon at 350 °C (with a heating rate of 3 °C/min, maintained for 10 min). The NPs were purified through three centrifugation steps (at 9960*g* for 10 min) using a mixture of chloroform, acetone, and methanol. Finally, the purified nanoparticles were redispersed in chloroform at a concentration of 10 mg/mL.

The LMNVs were synthesized using a lipid film hydration and ultrasonication method performed by optimizing a procedure reported in previous works of our group.^15^ Briefly, 18 mg of 1-stearoyl-*rac*-glycerol (GMS, Sigma-Aldrich), 2 mg of 1,2-dipalmitoyl-*rac-glycero*-3-phosphocholine (DPPC, Sigma-Aldrich), and 2 mg of methoxy-poly­(ethylene glycol)-1,2-distearoyl-*sn-glycero*-3-phospho-ethanolamine (mPEG-DSPE, 5000 Da, Nanocs) were dissolved in 500 μL of a 10 mg/mL IONPs solution in chloroform and heated to 70 °C to create a homogeneous lipid/IONP mixture. Separately, 3 mL of a 1 wt % aqueous solution of Tween 80 (Sigma-Aldrich), preheated to 70 °C, was added dropwise to the melted lipid/IONP mixture. The resulting dispersion was then vortex mixed for 2 min, followed by ultrasonication at 90% amplitude for 15 min using an ultrasonic probe (Fisherbrand Q125 Sonicator). After sonication, the LMNV suspension was cooled to 4 °C for 30 min and then purified by three successive centrifugation cycles at 16,000*g* for 90 min at 4 °C to remove excess surfactants and unencapsulated components. Finally, the purified LMNVs were redispersed in Milli-Q water.

Transmission electron microscopy (TEM) was performed to analyze morphology and size of IONPs. Before the assessment, the sample was sonicated at 8 W for 2 min with a ultrasonic probe (Bandelin). The images were acquired using a JEOL JEM1011 transmission electron microscope equipped with a thermionic electron source (tungsten) and operating at 100 kV on single-tilt sample holder. A drop of the sample dispersion was placed on a Cu grid, coated with an ultrathin amorphous carbon film, previously plasma treated (O_2_ + Ar plasma, 10 W, 2 min) to remove hydrocarbon residues. To enhance the contrast of the lipid component, the procedure included a 60 s negative staining using uranyl acetate solution (1% v/v).

Scanning electron microscopy (SEM) was performed to analyze the morphology and the size of the LMNVs. Drops of the dispersion of LMNVs in Milli-Q water (2 μL, 100 μg/mL) were placed on a silicon substrate and left to dry for 2 h at room temperature. Then, the samples were Au-sputtered using a Quorum Tech Q150RES coater (30 mA for 30 s). The SEM images were acquired using a dual-beam SEM system (Helios NanoLab 600i FIB/SEM, FEI).

Dynamic light scattering (DLS) measurements were conducted to evaluate the colloidal stability using a Zetasizer NanoZS90 (Malvern Instruments), to assess the hydrodynamic diameter, the polydispersity index (PDI), and the ζ-potential of LMNVs. Analyses were performed on LMNV samples (50 μg/mL) previously sonicated (10 W for 1 min at 25 °C) to obtain a homogeneous distribution, considering as dispersant Milli-Q water at 37 °C. Disposable polystyrene cuvettes (Malvern Zetasizer, Nano series) were used to measure the hydrodynamic diameter, while disposable folded capillary cells (Malvern Zetasizer, Nano series) were used for measuring ζ-potential.

Fourier-transformed infrared spectroscopy (FT-IR) was performed to identify the characteristic peaks of components related to IONPs and LMNVs, mainly related to the lipid matrix. The measurements were performed using a Shimadzu Miracle 10 on freeze-dried samples in the range of 500–4000 cm^–1^ with a resolution of 2 cm^–1^.

X-ray photoelectron spectroscopy (XPS) was carried out to identify the elements at the sample surface and their electronic states. A Kratos Axis Ultra^DLD^ spectrometer (Kratos Analytical Ltd.) was used, with a monochromated Al Kα X-ray source (*h*ν = 1486.6 eV) operated at 20 mA and 15 kV. Both IONPs and LMNVs samples were prepared by drop-casting a few microliters of an aqueous suspension on an indium substrate. The wide scans were collected over an analysis area of 300 × 700 μm^2^ at a photoelectron pass energy of 160 eV and with energy steps of 1 eV, while high-resolution spectra were collected at a photoelectron pass energy of 20 eV and energy steps of 0.1 eV. Charging effects on the surface of the samples were neutralized during the measurements using the Kratos charge neutralizer system. The obtained spectra were analyzed using CasaXPS software (Casa Software Ltd., version 2.3.24).

The thermogravimetric analysis (TGA) was conducted on both IONPs and LMNVs. TGA was performed on 5 mg of freeze-dried samples, using a Q500 analyzer from TA Instruments. The scans were performed in the range of 30–600 °C, using a 10 °C/min heating rate. Cooling was achieved using a 50 mL/min nitrogen flow.^16^

To assess the magneto-thermal properties of LMNVs and IONPs, the specific absorption rate (SAR) was determined by measuring the temperature rise of nanoparticle suspensions under AMF. Temperature variations were recorded using an OSENSA single-channel optic fiber in 100 μL of sample. The samples were placed in NMR tubes, positioned at the center of a 17-turn, 56 mm coil within a MagneTherm device (NanoTherics), and exposed to an AMF of 97.6 kHz and 20 mT. The LMNVs were resuspended in Milli-Q water (3 mg/mL) and the temperature was monitored for 15 min under AMF stimulation (AMF ON), followed by 10 min poststimulation (AMF OFF). The IONPs were resuspended in hexane (3 mg/mL) and the temperature was recorded for 5 min during AMF ON and 5 min poststimulation (AMF OFF) due to the different solvent properties. Magnetic heating properties were evaluated in 100 μL of nanoparticles suspension. The resulting temperature profiles were analyzed by measuring the specific absorption rate (SAR, eq 1) and the intrinsic loss power (ILP, eq 2) using the “corrected slope model” proposed by Wildeboer et al.^17^ In eq 1, *C*_S_ is the specific heat capacity of the solvent (J/K mL), *T* is the temperature (K), *L* is the linear loss parameter (W/K), and *c* is the nanoparticle concentration (mg/mL), with SAR in W/g; in eq 2 the SAR is expressed in W/kg, *f* is the frequency of the magnetic field (kHz), and *H* is the magnetic field strength (kA/m) to express ILP with the more convenient units of Hm^2^/kg.

$$\mathrm{SAR}=\left(C_{S}\frac{dT}{dt}+L\Delta T\right)/c$$ 1

$$\mathrm{ILP}=\frac{\mathrm{SAR}}{fH^{2}}$$ 2

### 2.2 Cell Cultures and Nanoparticles/Cells Interaction Investigations

Human microglia HMC3 cells (CRL-3304, ATCC) were cultured in minimum essential medium (MEM, Gibco) supplemented with 1% l-glutamine (100×, 200 mM, Gibco), 1% penicillin-streptomycin (100 IU/mL penicillin and 100 μg/mL streptomycin, Gibco), and 10% heat-inactivated fetal bovine serum (FBS, Gibco).

Human immortalized glioblastoma U87-MG cells (HTB-14, ATCC) were maintained in high-glucose Dulbecco’s modified Eagle’s medium (DMEM, Gibco) with 1% l-glutamine (100×, 200 mM, Gibco), 1% penicillin-streptomycin (100 IU/mL penicillin and 100 μg/mL streptomycin, Gibco), and 10% heat-inactivated FBS (Gibco).

Patient-derived GBM cells were obtained from resected tumor tissues of five patients diagnosed with grade IV primary glioma, IDH-1 wild-type, at San Martino Hospital (Genova, Italy). Samples were collected immediately after surgery with informed written consent, following ethical guidelines approved by the local ethics committee (*Registro CER Liguria* 341/2019). The cells were maintained in DMEM/F-12 (Gibco) with 1% l-glutamine (100×, 200 mM, Gibco), 1% penicillin-streptomycin (100 IU/mL penicillin and 100 μg/mL streptomycin, Gibco), and 10% heat-inactivated FBS (Gibco).

All cells were maintained until 70% confluency and used up to passage 12. For subculturing, 0.05% trypsin-EDTA (1×, Gibco) and phosphate-buffered saline (PBS, without Mg^2^^+^ and Ca^2^^+^, EuroClone) were used to detach and rinse the cells. For colorimetric assays and nanoparticles internalization, the cells were cultured with a phenol red-free DMEM supplemented with 25 mM HEPES (Gibco), 1% penicillin-streptomycin (P/S, EuroClone), 1% l-glutamine (Gibco), and 10% FBS (EuroClone) to minimize interferences in downstream analyses. Cell cultures were maintained in sterile condition in an incubator at 37 °C in a humidified atmosphere with 5% CO_2_.

The biocompatibility of LMNVs was evaluated on HMC3 cells by cell viability assays, including WST-1 assay, Qant-iT PicoGreen dsDNA assay, and the LIVE/DEAD assay.

Cell viability was assessed using the WST-1 Assay Reagent—Cell Proliferation (Abcam), which measures metabolic activity as an indicator of cell proliferation and viability. HMC3 cells were seeded at 1.5 × 10^4^ cells/cm^2^ in 96-well plates and incubated with increasing concentrations of LMNVs (0, 10, 25, 50, 100, and 250 μg/mL) or IFN-γ (ThermoFisher), as a positive control, at 0.6 μg/mL for 24 and 72 h at 37 °C, 5% CO_2_. After the treatment, WST-1 reagent was added 1:11 in the phenol red-free complete DMEM to each well and incubated for 1.5 h at 37 °C, 5% CO_2_. The supernatant was recovered and transferred to a reading 96-well plate (Corning). The absorbance was measured at 450 nm using a PerkinElmer Victor X3 UV–Vis spectrophotometer. The results were normalized to untreated controls and expressed as a percentage of metabolic activity.

The Quant-iT PicoGreen dsDNA Assay Kit (Invitrogen) was used to quantify double-stranded DNA (dsDNA) as a measure of cell viability and proliferation. HMC3 cells were seeded in a 96-well plate (Corning) at a density of 1.5 × 10^4^ cells/cm^2^ in phenol red-free complete DMEM. After 24 h, the cells were treated with increasing concentrations of LMNVs (0, 10, 25, 50, 100, and 250 μg/mL) and IFN-γ at 0.6 μg/mL as a positive control. After 24 and 72 h of incubation at 37 °C, 5% CO_2_, cells were washed with PBS, resuspended in 100 μL of Milli-Q water, and subjected to three freeze–thaw cycles (−80 to 37 °C) to induce lysis and release dsDNA. The assay was performed following the manufacturer’s procedure. Briefly, 40 μL of assay buffer, 30 μL of cell lysate, and 30 μL of PicoGreen reagent were mixed in a black polystyrene 96-well plate (Corning Costar). After 5 min of incubation at room temperature in the dark under shaking conditions, fluorescence was measured using a VICTOR X3 plate reader at λ_ex_ = 485 and λ_em_ = 535 nm. The results were normalized to untreated controls and expressed as a percentage of cell viability.

Cell viability and membrane integrity were assessed using the LIVE/DEAD Cell Viability Assay Kit (Thermo Fisher Scientific) to determine the percentage of viable cells relative to the total population. HMC3 cells were seeded at 1.5 × 10^4^ cells/cm^2^ in 24-well μ-Plate (Ibidi) with complete MEM and incubated for 24 h. Following cell adhesion, cultures were treated with increasing concentrations of LMNVs (10, 25, 50, 100, and 250 μg/mL). IFN-γ at 0.6 μg/mL was included as a positive control. After 72 h of incubation, the cells were washed with PBS and incubated for 30 min with phenol red-free DMEM containing 5 μg/mL of Hoechst (Invitrogen) to stain nuclei, 4 μM ethidium homodimer-1 (ThermoFisher) to stain dead cells, and 2 μM calcein-AM (Fisher Scientific) for live cells. Following staining, the cells were washed with PBS and imaged using a confocal laser scanning microscope with a Plan Fluor 10×/0.30 objective (C 2s system, Nikon). For quantitative analysis, the number of dead cells was counted relatively to the total cell population using ImageJ software, and the ratio of the LIVE/DEAD cells was calculated accordingly.

The internalization and intracellular distribution of LMNVs in HMC3 cells were assessed at three different time points (4, 24, and 72 h) using flow cytometry for quantitative analysis and confocal microscopy for cellular localization. Analyses were conducted using LMNVs at a concentration of 100 μg/mL, labeled with the fluorescent dye DiO. For nanoparticle labeling, DiO (Vybrant Multicolor Cell-Labeling Kit, Thermo Fisher) was added at a 1:100 dilution to a 1 mg/mL LMNV suspension in Milli-Q water. The mixture was stirred in the dark at room temperature for 1 h. To remove excess dye, the solution underwent two centrifugation cycles at 16,000*g* for 1.5 h at 4 °C, followed by resuspension in Milli-Q water.

Flow cytometry was performed to quantify LMNV uptake over time. HMC3 cells were seeded in 24-well plates (Corning) at a density of 1.5 × 10^4^ cells/cm^2^ in 500 μL of MEM and incubated for 24 h. Following adhesion, cells were treated with DiO-labeled LMNVs (100 μg/mL) dispersed in phenol red-free complete DMEM for 4, 24, and 72 h. At the end of each incubation period, cells were detached using 0.05% trypsin-EDTA, resuspended in PBS, and analyzed for fluorescence intensity (λ_ex_ = 490, λ_em_ = 565 nm) using a CytoFLEX flow cytometer (Beckman Coulter). Data were processed with CytExpert software (Beckman Coulter), where fluorescence thresholds were established with respect to untreated control cells to quantify nanoparticle uptake.

The subcellular localization of LMNVs was assessed using confocal laser scanning microscopy (C 2s, Nikon). HMC3 cells were seeded at 1.5 × 10^4^ cells/cm^2^ in Willco Petri dishes (GWST-3512) and treated as previously described with DiO-labeled LMNVs, followed by incubation for 4, 24, and 72 h. At each time point, cells were washed with PBS and stained in phenol red-free DMEM containing 5 μg/mL Hoechst (Invitrogen) for nuclear staining, 5 μM LysoTracker-Red (ThermoFisher) for lysosomal imaging, and 0.05 μM tetramethylrhodamine methyl ester (Life Technologies) for mitochondrial imaging. After an incubation at 37 °C for 30 min, cells were washed with PBS, and fresh phenol red-free DMEM was added before imaging. Confocal images were acquired using the Nikon C 2s microscope and analyzed with Nikon View Software to determine Pearson’s coefficient, evaluating the colocalization of LMNVs within lysosomes and mitochondria.

### 2.3 Microglia Stimulation

To ensure optimal exposure of microglia to AMF and induce their polarization into the M1-like phenotype, a custom cell culture substrate was fabricated using poly­(dimethylsiloxane) (PDMS) and coverslip glass. Briefly, glass substrates bonded with a PDMS frame were fabricated to optimize cell culture conditions. Sylgard 184 silicone elastomer (DOW) was prepared in a 1:10 elastomer-to-cross-linker ratio, mixed thoroughly, and degassed. The mixture was then cured at 70 °C for 2 h and cut into 2.4 cm^2^ squares. Both the PDMS and glass slides were cleaned, treated with O_2_ plasma (40 W, 0.50 mbar, 15 s) and bonded by heating at 70 °C for 30 min to ensure a stable and irreversible attachment. Finally, the substrates underwent UV sterilization and were pretreated with MEM overnight to increase the cell adhesion before cell seeding.

Calcium imaging was performed to analyze intracellular Ca^2^^+^ levels during acute AMF stimulation. HMC3 cells were seeded in a Willco Petri dish (GWST-3512) at a density of 1.5 × 10^4^ cells/cm^2^ in complete MEM and incubated overnight. At 24 h of culture, cells were maintained in complete phenol red-free DMEM and treated with or without 100 μg/mL of LMNVs. At 24 h of incubation, cells were stained with Fluo-4 AM (1 μM, Invitrogen) for 30 min at 37 °C, rinsed with PBS, and incubated in phenol red-free DMEM to perform fluorescence time-lapse microscopy experiment during AMF stimulation. The control group without LMNVs (AMF) and LMNV-treated cells were exposed to an AMF using a MagneTherm system equipped with the Live Cell Exposure Option (NanoTherics), which allows for direct exposure of samples on a microscope stage at 20 mT, 214 kHz. The analysis was performed with AMF off for 900 s and with AMF on for 3600 s. Time-lapse images were acquired from different fields every 12 min using a 20× objective with the Nikon C 2s confocal microscope. To investigate the contribution of extracellular versus intracellular Ca^2+^ during magneto-thermal stimulation, LMNV-treated cells were exposed to AMF in calcium-free phosphate-buffered saline. Prior to AMF exposure, cells were washed three times with Ca^2+^-free solution to ensure complete removal of extracellular calcium, and imaging was performed under the same conditions described above.

Concerning chronic stimulation of microglia to AMF, HMC3 cells were seeded on the fabricated cell culture substrates at a density of 1.5 × 10^4^ cells/cm^2^ in complete MEM and incubated overnight. After 24 h, the cells were treated according to five experimental groups: the control group (CTRL), which consisted of cells maintained in complete phenol red-free DMEM (untreated cultures), the AMF group, where cells were cultured under the same conditions as the CTRL group but exposed to AMF, the LMNVs group, where cells were treated with 100 μg/mL of LMNVs in complete phenol red-free DMEM, the LMNVs + AMF group, where cells were cultured under the same conditions as the LMNVs group and subjected to AMF stimulation, and eventually the group treated with human recombinant interferon-γ (IFN-γ group), which served as the positive control for M1 microglia activation, where cells were treated with 0.6 μg/mL of IFN-γ for 3 days. For the AMF exposure, the cells were placed inside a 17-turn, 56 mm coil of a MagneTherm device (NanoTherics) equipped with a 200 nF capacitor and exposed to an AMF of 20 mT at 97.6 kHz, maintaining a temperature of 37 °C. The cells were exposed to the AMF for 2 h per day for 3 consecutive days. In parallel, non-AMF-treated samples were incubated at 37 °C outside the CO_2_-controlled incubator during the stimulation period, ensuring that the conditions for these samples were consistent with those of the AMF-exposed cells.

To evaluate the expression of M1-like microglia phenotype markers, the presence of CD40 and CD86 was assessed through flow cytometry analysis using the CytoFLEX cytometer (Beckman Coulter). At the end of the chronic AMF treatment, cells were detached and the pellet was resuspended in 400 μL of 4% paraformaldehyde (PFA) in PBS and fixed in suspension at 4 °C for 20 min, followed by centrifugation at 860*g* for 10 min at 4 °C. Samples were then resuspended in 400 μL of PBS containing 10 μg/mL of either FITC anti-CD40 or FITC anti-CD86 antibody (λ_ex_ = 493, λ_em_ = 528 nm, Abcam) for 30 min at 4 °C in the dark, cells were centrifuged again (860*g*, 4 °C, 10 min) and resuspended in PBS Data acquisition and analysis were performed with CytExpert software (Beckman Coulter).

After chronic exposure to AMF, morphological changes were observed in HMC3 cells. These changes were assessed using a Nikon C 2s confocal microscope equipped with a 60× oil immersion objective. The cells were fixed with 4% w/v PFA with incubation at 4 °C for 20 min and then washed with PBS three times. Thereafter, cultures were treated with Triton X-100 (0.1%, v/v, in PBS) for 30 min at room temperature. Following the removal of the Triton X-100 solution, the cells were treated with goat serum (10% v/v in PBS) for 40 min at room temperature to reduce nonspecific backgrounds, and then incubated for 1 h at room temperature with a goat serum solution containing 5 μg/mL of Hoechst (Thermo Fisher Scientific) to label the nuclei and 2.5 μg/mL of TRITC-phalloidin (Sigma) to label the actin filaments of the cytoskeleton. Following incubation, the cells were rinsed with PBS, and fresh PBS was added to perform confocal observation. The quantitative analysis was conducted using the ImageJ-Fuji extension, considering circularity and aspect ratio as parameters.

To evaluate the mRNA expression of IL-6, IL-8, and TNF-α, quantitative real-time reverse-transcription polymerase chain reaction (qRT-PCR) analysis was performed in all experimental classes after chronic AMF stimulation. mRNA was isolated and purified using the RNeasy Mini Kit (Qiagen) following the manufacturer’s guidelines. RNA concentration and purity were assessed using a NanoDrop spectrophotometer (Thermo Fisher Scientific), where 1 μL from each sample was analyzed. Reverse transcription was carried out using 5 ng of RNA from each experimental class using the iScript Advanced cDNA Synthesis Kit for qRT-PCR (Bio-Rad) to obtain complementary DNA (cDNA). The thermal cycling conditions for reverse transcription were 46 °C for 20 min, followed by 95 °C for 1 min. Amplification of the cDNA was performed using the SsoAdvanced Universal SYBR Green Supermix (Bio-Rad) and the CFX Connect Real-Time PCR Detection System (Bio-Rad). The amplification program consisted of an initial step at 95 °C for 30 s for polymerase activation and DNA denaturation, followed by 50 cycles at 98 °C for 10 s and at 60 °C for 20 s. At the end of the amplification cycles, a temperature ramp from 65 to 95 °C with 0.5 °C/s increments was carried out to obtain melting curves and confirm the specificity of the qRT-PCR amplification. Gene transcription levels were normalized to the housekeeping gene 18S rRNA, and the ΔΔCt relative expression was finally calculated. Gene modulation >5-fold or <0.05-fold, along with *p* < 0.05, was used to determine upregulation and downregulation.

To evaluate the secretion of inflammatory cytokines after chronic AMF stimulation, the cell supernatant from each experimental group was collected, centrifuged at 21,000*g* for 10 min, and analyzed using ELISA. The concentration of IL-6, IL-8, and TNF-α were quantified using specific ELISA kits from Invitrogen according to the manufacturer’s instructions; to prevent signal saturation, the cell supernatants were diluted 1:100 for IL-6, 1:100 for IL-8, and 1:2.5 for TNF-α in phenol red-free DMEM. The final cytokine concentrations were then calculated by applying the corresponding dilution factors. The measurements were performed using a Victor X3 Plate Reader, by measuring absorbance at 450 nm.

To evaluate more in detail the effects on microglia of the different treatments, a transcriptomic analysis was also performed. Total RNA was extracted using RNeasy Mini Kit (Qiagen) as described previously in this section, and samples were then processed by using the GeneWiz Ultra-Low Input RNA-Seq workflow (AZENTA—Life Science). Whole transcriptome sequencing libraries were prepared starting from the extracted RNA; rRNA (rRNA) was first depleted to enrich for nonribosomal transcripts. The RNA fraction was subsequently fragmented and subjected to random priming to ensure unbiased coverage across the transcriptome. First- and second-strand cDNA synthesis was then performed, generating double-stranded cDNA molecules. The cDNA ends were repaired, 5′-ends phosphorylated, and dA-tailed to allow adaptor ligation. Adaptors were ligated to both ends of the cDNA fragments, followed by PCR enrichment. The final libraries were then subjected to high-throughput sequencing. Sequence reads were trimmed to remove possible adapter sequences and nucleotides with poor quality using Trimmomatic v.0.36. The trimmed reads were mapped to the *Homo sapiens* GRCh38 reference genome available on ENSEMBL using the STAR aligner v.2.5.2b. Using DESeq2, a comparison of gene expression among the customer-defined groups of samples was performed. The Wald test was used to generate *p*-values and log2 fold changes; genes with an adjusted *p* < 0.05 and absolute log2 fold change >1 were considered as differentially expressed genes for each comparison.

### 2.4 Microglia-Mediated Effects on Glioblastoma Cells

The potential of M1-like microglia-based immunotherapy to reduce GBM cell proliferation and viability was assessed by evaluating the effects of conditioned medium from microglia treated under the different experimental conditions (CTRL, AMF, LMNVs, LMNVs + AMF, IFN-γ) both on patient-derived GBM cells and on the immortalized GBM cell line U87-MG. The microglia-conditioned medium was obtained following each treatment, centrifuged at 16,000*g* for 10 min at room temperature, and used to incubate GBM cells. Patient-derived GBM cells were seeded at a density of 9 × 10^3^ cells/cm^2^ in 96-well plates (Corning) and in μ-Plate 96-well plates. After 24 h, the culture medium was replaced with microglia-conditioned medium specific to each experimental condition, followed by a 4 day incubation. U87-MG cells were seeded at a density of 5 × 10^4^ cells/cm^2^ and treated under the same conditions. Cell viability was assessed using the LIVE/DEAD and Qant-iT PicoGreen assays, while metabolic activity was measured with the WST-1 assay, as previously described.

Patient-derived and U87-MG cells were also processed to monitor through immunofluorescence the expression of the *K*_i_-67 marker upon incubation with microglia-conditioned medium. Briefly, cells were fixed with 4% w/v PFA at 4 °C for 20 min and washed three times with PBS. Permeabilization was performed using 0.1% v/v Triton X-100 in PBS for 30 min at room temperature. Thereafter, cells were blocked with 10% v/v goat serum in PBS for 40 min at room temperature to minimize nonspecific binding. Subsequently, cells were incubated overnight at 4 °C with a rabbit antihuman *K*_i_-67 primary antibody (1:200, Sigma-Aldrich) diluted in 10% v/v goat serum. Following three PBS washes, cells were stained with a solution containing 10 μg/mL of a secondary antibody (F­(ab′)­2-goat anti-Rabbit IgG H + L Alexa Fluor 488 conjugate, Invitrogen), 5 μg/mL of Hoechst, and 2.5 μg/mL of TRITC-phalloidin in 10% v/v goat serum for 1 h at room temperature.

The same immunofluorescence staining protocol was used to evaluate the localization of HMGB1 using a rabbit HMGB1 polyclonal antibody (1:200, ThermoFisher), followed by a secondary antibody. Concerning CRT, the permeabilization step with 0.1% v/v Triton X-100 in PBS was shortened to 5 min to preserve plasma membrane integrity and to detect only surface-exposed CRT, which is a hallmark of ICD; a direct immunofluorescence approach has been thereafter followed, with a fluorescent anti-CRT antibody (Alexa Fluor 488 Anti-Calreticulin antibody [EPR3924]—ER Marker, 1:150, Abcam).

All Images have been acquired with a confocal laser scanning microscope equipped with a Plan Fluor 60× oil immersion objective (C 2s system, Nikon). The percentage of the cells positive for marker expression was calculated and analyzed with the ImageJ-Fiji extension software for both *K*_i_-67 and CRT, while for HGBM1 the analysis focused on evaluating the percentage of cells displaying translocation of the marker from the nucleus to the cytoplasm.

### 2.5 Statistical Analysis

Data normality was assessed with the Shapiro–Wilk test. For normally distributed data, one-way ANOVA was conducted, followed by LSD post hoc analysis with Bonferroni’s correction, and results were expressed as mean ± standard error. Non-normally distributed data were analyzed using the Kruskal–Wallis test, followed by pairwise Wilcoxon post hoc analysis with Holm’s correction, and results were reported as median ± 95% confidence interval. Statistical analyses were performed using *R* software. The significance was set at *p* < 0.05, and data were presented as the mean value ± standard deviation; unless otherwise specified, all experiments were performed at least in triplicate.

## 3 Results and Discussion

### 3.1 Nanoparticle Characterization

The morphology and size of the synthesized IONPs were examined using TEM. The IONPs were found to be predominantly spherical with an average diameter of 18 nm (Figure 1a), owning a homogeneous size distribution, as shown in a representative TEM image (Figure 1b).

**Figure 1:**
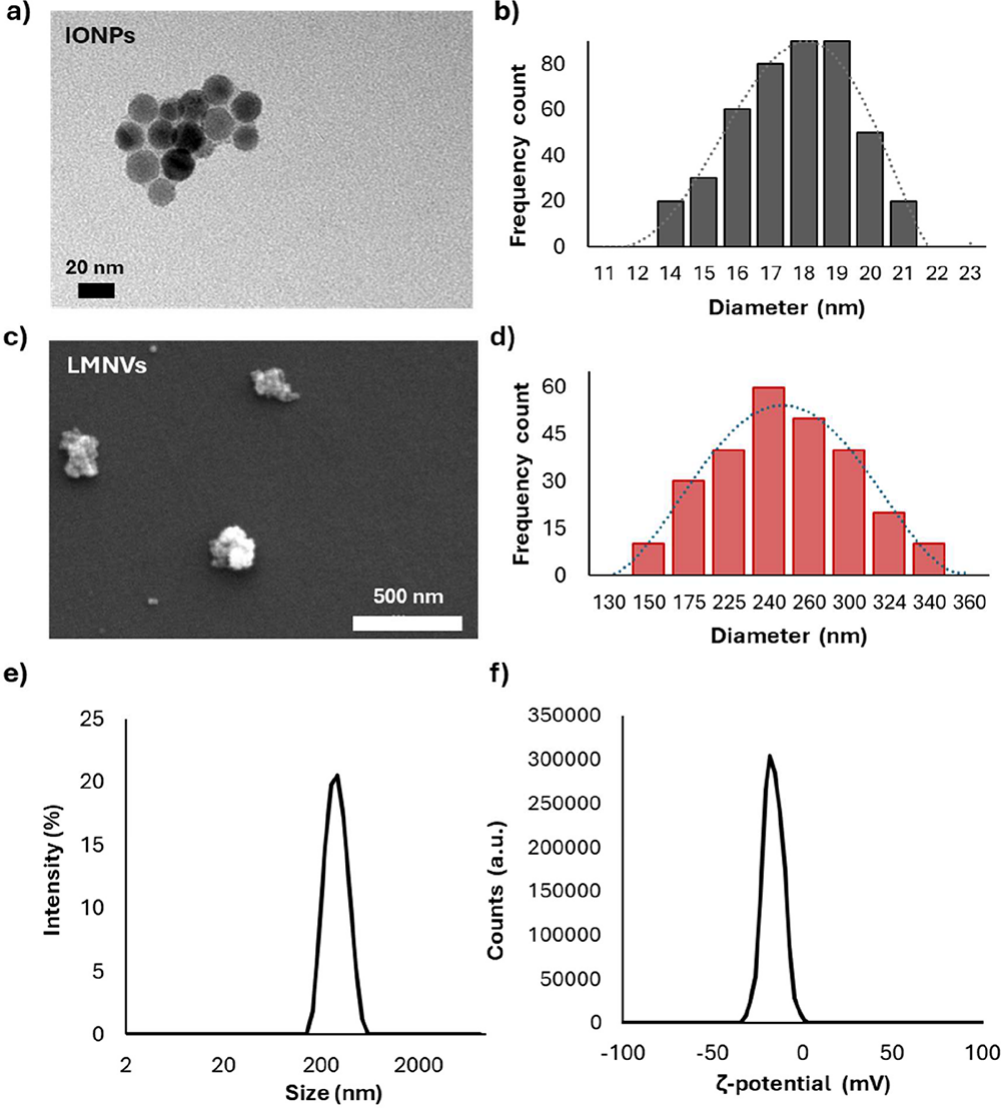
Nanoparticle morphology and size. Representative TEM image of IONPs (a); size distribution of IONPs obtained from TEM images (b); representative SEM image of LMNVs (c); size distribution of LMNVs obtained from SEM images (d); DLS intensity distribution (%) as a function of hydrodynamic diameter (nm) of LMNVs (e); and ζ-potential distribution (a.u.) as a function of ζ-potential (mV) of LMNVs (f).

The morphology of LMNVs encapsulating IONPs was analyzed using SEM and TEM. The LMNVs displayed a blackberry-like structure, as illustrated in the representative SEM image (Figure 1c), with an average diameter of 260 nm, as shown in Figure 1d. The TEM analysis confirmed the average diameter, as reported in Figure S1. DLS analysis of LMNVs showed an average hydrodynamic diameter of 282 ± 5 nm, indicating a monodisperse size distribution with a polydispersity index of 0.145 ± 0.019 (Figure 1e). The ζ-potential resulted −17.90 ± 0.43 mV (Figure 1f), suggesting a satisfactory stability in an aqueous medium at 37 °C.

FT-IR spectroscopy was performed to identify the chemical bonding and interactions between IONPs and the lipid matrix in LMNVs, as well as to confirm the presence of IONPs in LMNVs. As illustrated in Figure 2a, a comparison of the FT-IR spectra for both IONPs and LMNVs shows a characteristic Fe–O vibration peak within the range of 570–630 cm^–1^, which is attributed to the presence of the encapsulated IONPs.^18^ Additionally, a peak between 1000 and 1200 cm^–1^ is observed, corresponding to the P–O stretching vibrations, which is indicative of the presence of phospholipids such as DPPC, the primary lipid component of the lipid matrix in LMNVs.^15^ Moreover, the spectrum also displays a band between 2800 and 3000 cm^–1^, which can be attributed to the stretching vibrations of the CH_2_ and CH_3_ groups in the lipid hydrocarbon chains.^15^

**Figure 2:**
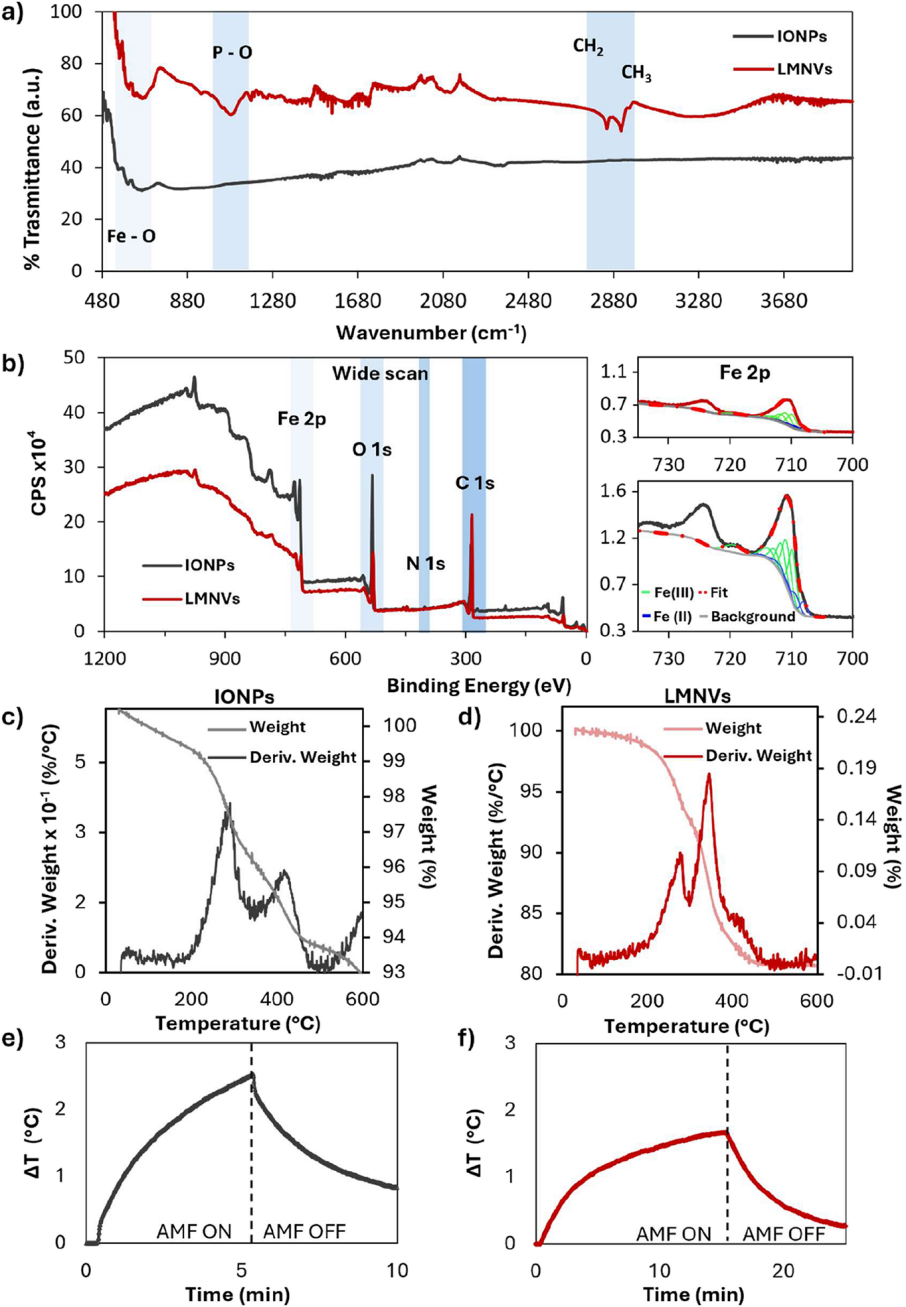
Nanoparticle chemical and physical characterization. FT-IR spectra of IONPs (gray) and LMNVs (red) (a); XPS-wide scan of IONPs (gray) and LMNVs (red) displaying the corresponding peaks for Fe 2p, O 1s, N 1s, and C 1s, with high-resolution spectrum for Fe 2p (b); TGA graphs presenting the weight reduction of IONPs (c) and LMNVs (d); and temperature profile over time for a 3 mg/mL IONP dispersion in hexane (e) and for a 3 mg/mL LMNVs dispersion in Milli-Q water (f) during the stimulation with AMF at 20 mT and 97.6 kHz (AMF ON) and after the end of the stimulus (AMF OFF).

XPS was performed to obtain a detailed characterization of the chemical functional groups constituting IONPs and LMNVs. The XPS-wide scans, which exhibit common signals in both spectra related to Fe 2p, O 1s, N 1s, and C 1s, are illustrated in Figure 2b. The analysis revealed that the surface of IONPs contains 9.8% iron (Fe), 33.6% oxygen (O), and 54.5% carbon (C), with slight traces of chlorine (Cl). The significant presence of carbon is consistent with the stabilization by oleic acid.^19^ The observed O/Fe ratio (close to 3.4), higher than expected for pure iron oxides, suggests that the O-containing organic ligands effectively coated the inorganic core, reducing direct exposure to the iron oxide surface. The slight chlorine (Cl) traces can be attributed to residual chloroform from the dispersion process. In contrast, the surface composition of LMNVs consists of 3.5% iron (Fe), 19.9% oxygen (O), and 76.0% carbon (C). The increase of oxygen and carbon can be related to the organic components associated with the lipid matrix.

Focusing on the binding energy region between 700 and 740 eV, characteristic of the Fe 2p peaks, we can identify Fe 2p_3/2_ and Fe 2p_1/2_ at approximately 710–715 and 725–730 eV, respectively.^20^ For both samples, the Fe 2p spectrum also shows a low-intensity satellite centered at approximatively 719 eV, typically observed in Fe­(III) compounds but absent in the case of pure Fe_3_O_4_.^21^ To get more insights into the Fe­(II) and Fe­(III) contents of the two samples, fitting of the obtained XPS spectra is needed. It has to be taken into account that, as in the case of most of the transition metals, the XPS signal undergoes the so-called multiplet splitting, and multiple peaks are needed to correctly describe a single chemical state.^22^ Based on the work of Biesinger et al.,^22^ the Fe 2p signals collected on IONPs and LMNVs can be described as originated from a mixed Fe_2_O_3_/FeO system, with Fe­(III) being approximately the 80–85% of the total Fe content. The results of the best fit procedure are reported in the right part of Figure 2b. The oxygen signal can be attributed to O–Fe groups (either Fe­(II) or Fe­(III)), hydroxide or defective oxides, organic oxygen, and adsorbed water. An important observation to note is that the intensity of the organic oxygen component of LMNVs significantly increased compared to the IONPs, as shown in Figure S2a,b, due to the presence of the lipidic matrix. The presence of oxygen and carbon-bonded organic components in LMNVs is indicative of a good interaction between IONPs and the lipid matrix: XPS analysis thus confirms the encapsulation of IONPs in the lipid matrix, evidenced by the increased presence of organic components and modified surface interactions.

To detect the percentage of iron and lipid/polymeric components, TGA was performed. The curves of both IONPs and LMNVs display a progressive decrease in total weight as temperature increases, providing insight into the composition and thermal stability of the samples. The TGA curve of IONPs exhibits minimal weight loss due to the oleic acid coating, with only a 5.6 wt % reduction observed above 450 °C (Figure 2c). In contrast, for LMNVs, the TGA curve (Figure 2d) shows an initial weight loss of 5.84 wt %, likely due to the evaporation of absorbed water or the presence of residual surfactants on the surface. A second, more significant weight loss of 12.40% occurs at higher temperatures, corresponding to the thermal degradation of lipidic and polymeric components, specifically GMS, mPEG-DSPE, and DPPC. Beyond 450 °C, no further weight loss is observed, indicating the complete degradation of the organic matrix. The remaining mass, accounting for approximately 81 wt % of the initial weight, corresponds to the IONPs encapsulated within the LMNVs. Overall, TGA analysis confirms that LMNVs are composed of approximately 81 wt % IONPs, with the remaining 19 wt % attributed mainly to the lipidic matrix.

To assess the potential of IONPs and LMNVs for inducing magnetic hyperthermia under AMF stimulation, the nanoparticles were exposed to a magnetic field strength (*H*) of 20 mT at 97.6 kHz of frequency (*f*). The AMF parameters selected are three times higher than the Brezovich’s limit defined as *H* × *f* and around 5 × 10^8^ A m^–1^ s^–1^, which is traditionally considered the safety threshold to prevent adverse effects from eddy currents.^23^ However, recent in vivo studies suggest that tolerance limits may be increased up to 10-fold without causing significant physiological harm.^24^ Consequently, the parameters used in this study are within the updated safety limits based on these more recent findings.^25^

The evaluation of the heating capacity of LMNVs upon the stimulation with AMF is essential, particularly concerning the impact of the lipid matrix on this property. Figure 2e,f present the magnetic heating properties of the nanoparticles, illustrating the temperature variations over time during and after AMF exposure for IONPs and LMNVs. As shown in Figure 2f, LMNVs in water exhibit similar heating behavior of IONPs in hexane, with an increase of temperature respectively below 3 and 2 °C; however, the temperature increase in LMNVs occurs at a slower rate with respect to IONPs: this discrepancy can be likely attributed to the increased viscosity introduced by the lipid matrix, a phenomenon also reported in other studies that observed a correlation between a reduced heating rate and a lower concentration of ferromagnetic elements in nanoparticle compositions.^26^ The slight temperature increase in LMNVs is useful in all those applications where you do not want to damage the cell but, as in this case, induce a modulation of the phenotype. This makes LMNVs a promising tool for applications such as immune cells, presenting a potential immunotherapeutic strategy distinct from traditional oncological hyperthermia.^27^

Despite the slight difference in heating kinetics, the quantitative assessment of SAR and ILP suggests that the lipid matrix has minimal influence on magnetic heating performance. IONPs exhibit a SAR of 11.19 ± 0.48 W/g, while LMNVs show a slightly lower SAR of 10.80 ± 0.22 W/g. This indicates that the lipid coating does not significantly affect the magnetic properties of the IONPs in terms of heating efficiency. Furthermore, the evaluation of ILP, a key parameter reflecting the overall heating efficiency of the nanoparticles, shows comparable values for both IONPs and LMNVs, respectively of 0.45 ± 0.01 nHm^2^/kg and 0.44 ± 0.09 nHm^2^/kg, supporting the conclusion that the lipid shell has a minimal impact on the magnetic heating performance of the nanoparticles. Based on these considerations, LMNVs can be considered particularly useful in applications where modulation of cellular behavior is relevant, without reaching temperature potentially harmful for the cells, such as indeed microglia immunomodulation.

### 3.2 Nanoparticle/Cell Interactions

The biocompatibility of LMNVs was assessed by evaluating metabolic activity through WST-1 assay, cell proliferation through PicoGreen assay, and cell viability through LIVE/DEAD assay following microglia treatment with different LMNV concentrations (0–250 μg/mL). After 24 h of incubation (Figure S3a), a significant reduction (*p* < 0.05) in metabolic activity was observed just at the highest concentration (250 μg/mL). At 72 h, a further significant reduction (*p* < 0.05) in metabolic activity was detected at 250 μg/mL, although a slight recovery was noted with respect to the 24 h time point (Figure S3a). Similar trends were observed for cell proliferation (Figure S3b) and viability (Figure S3c,d) at 72 h, thus suggesting 100 μg/mL as a safe working concentration; this was also confirmed by the representative confocal images (Figure S3c) and the relevant quantitative live/dead cell assessment (Figure S 3d).

The cell viability was also evaluated by comparing 50 and 100 μg/mL LMNV concentrations in combination with AMF stimulation to evaluate, after chronic stimulation, the combined effect of LMNVs and AMF (20 mT, 97.6 kHz, 2 h per 3 days). The data did not show any significant alteration of live cells out of dead cells among all the experimental groups (Figure S3e,f), confirming 100 μg/mL as a safe working concentration also in combination with AMF stimulation.

The internalization of LMNVs was assessed through confocal microscopy to determine their intracellular localization and through flow cytometry to quantify uptake over time, by using fluorescently labeled DiO-LMNVs. As shown in Figure S4a,b, confocal microscopy analysis revealed a time-dependent intracellular distribution of LMNVs. DiO-LMNVs were predominantly localized within lysosomes, as indicated by a progressive increase in the Pearson’s correlation coefficient between the nanoparticle and lysosome fluorescence signals: 0.135 ± 0.024 at 4 h, 0.383 ± 0.088 at 24 h, and 0.609 ± 0.031 at 72 h (Figure S4c). In contrast, colocalization with mitochondria remained minimal throughout the time course, with Pearson’s correlation coefficients of 0.012 ± 0.003 at 4 h, 0.070 ± 0.007 at 24 h, and 0.068 ± 0.009 at 72 h (Figure S4c).

The intracellular localization was also quantitatively analyzed by flow cytometry. As shown in Figure S4d,e, the percentage of DiO-LMNV-positive (LMNVs^+^) cells was determined relative to the fluorescence threshold established from untreated control cells (CTRL). At 4 h postincubation, only a small fraction of cells (19.6 ± 0.6%) exhibited LMNV uptake. However, a substantial increase in internalization was observed after 24 h, with 94.0 ± 0.9% of cells showing fluorescence, indicating a marked enhancement in nanoparticle internalization. By 72 h, nearly all cells (98.2 ± 0.2%) had internalized LMNVs, confirming highly efficient cellular uptake. These results support the notion that LMNVs exhibit excellent internalization efficiency, likely facilitated by the lipid coating. In addition, prevalent lysosomal localization could drive LMNV degradation in these organelles, with and a potential involvement in the iron cycle.^28^ This efficient uptake profile is particularly relevant for therapeutic applications, as it ensures widespread nanoparticle delivery to target cells, which could influence downstream cellular responses, especially in combination with external stimuli, including microglial polarization.

### 3.3 Nanoparticles-Mediated Hyperthermal Stimulation

The ability of LMNVs to trigger a response in cells upon AMF application was assessed first with an acute stimulation by exploiting Ca^2^^+^ imaging. As shown in Figure 3a, AMF stimulation of untreated HMC3 control cells (AMF group) did not result in any measurable change fluorescence, indicating the absence of intracellular Ca^2^^+^ level alterations. Conversely, AMF stimulation of HMC3 cells preincubated with LMNVs (LMNVs + AMF group) led to a continuous and sustained increase in fluorescence over time (Figure 3b), reflecting a progressive rise in intracellular Ca^2^^+^ levels. This Ca^2^^+^ influx, observed exclusively in the presence of both LMNVs and AMF stimulation, suggests that the magneto-thermal conversion effect of LMNVs plays a key role in cellular activation. Representative time-lapse confocal images are reported in Figure 3b. These results suggest that the adopted stimulation approach can induce intracellular Ca^2^^+^ mobilization, likely as a result of localized temperature changes at the nanoparticle-cell interface. The elevation in intracellular Ca^2^^+^ levels is a well-known trigger for various signaling pathways involved in microglial activation and polarization, particularly toward an M1-like phenotype.^29^ The obtained data thus provide strong evidence supporting the functional impact of LMNV-mediated magneto-thermal stimulation on microglial polarization dynamics.

**Figure 3:**
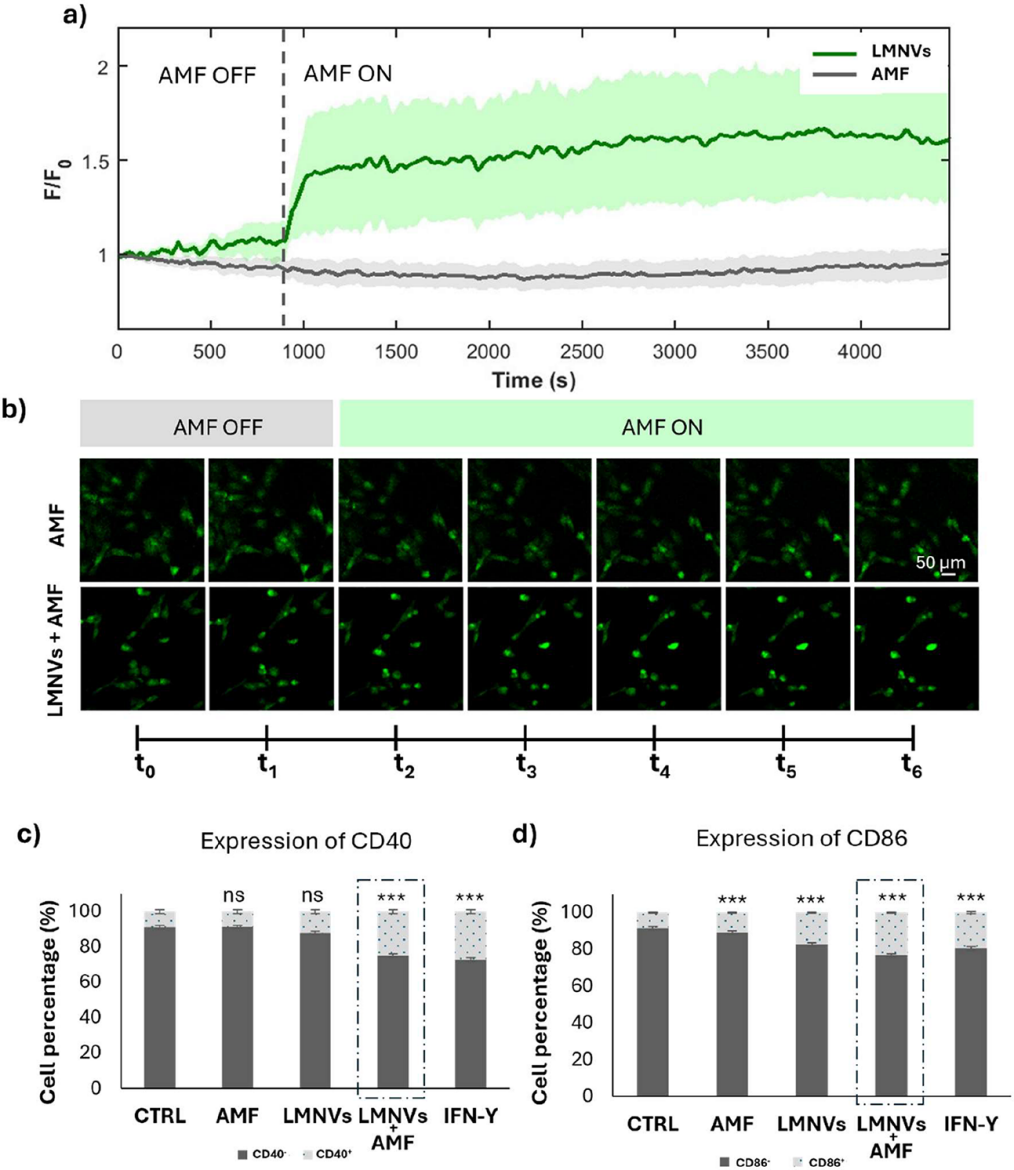
Acute and chronic stimulation. Cell fluorescence levels (*F*/*F*_0_) indicative of Ca^2+^ concentrations over the time during AMF stimulation in microglia cells or without LMNVs (a); representative confocal images of cell of Ca^2+^ at different time-dependent frames extracted every 12 min (*t*_0_–*t*_6_) (b); and CD40 (c) and CD86 (d) marker expression after chronic AMF stimulation (ns *p* > 0.05, *** *p* < 0.001).

To further elucidate the origin of the Ca^2^^+^ signal induced by LMNV-assisted magneto-thermal stimulation, an experiment was conducted in the absence of extracellular Ca^2^^+^. As shown in the representative time-lapse confocal images (Figure S5a), under extracellular Ca^2^^+^-free conditions AMF stimulation of LMNV-treated HMC3 cells did not result in a significant increase in average intracellular Ca^2^^+^ levels, as determined by normalized fluorescence intensity (*F*/*F*_0_, Figure S5b). The absence of a sustained elevation in cytosolic Ca^2^^+^ indicates that extracellular Ca^2^^+^ influx is required to maintain prolonged intracellular calcium signaling. However, time-lapse analysis revealed that a subset of cells exhibited transient increases in fluorescence during AMF application: these Ca^2^^+^ waves, although not reflected in the population-average trace, suggest a partial contribution from intracellular calcium stores (e.g., endoplasmic reticulum). Therefore, while the observed transient responses suggest that intracellular stores can initiate Ca^2^^+^ mobilization upon magneto-thermal stimulation, sustained signaling depends on the availability of Ca^2^^+^. These findings partially align with previous observations by Zhu et al.,^30^ who demonstrated that photothermal stimulation can induce Ca^2^^+^ release from intracellular stores, such as the endoplasmic reticulum; moreover, Nadezhdin et al.^31^ have shown that heat can activate ion channels in the plasma membrane of microglia, facilitating calcium influx from the extracellular environment. Our observations, therefore, indicate that both intracellular release and extracellular influx contribute to the Ca^2^^+^ dynamics during magneto-thermal stimulation.

The hypothesis that AMF stimulation enhances the inflammatory response of microglia was investigated through immunocytochemistry against CD-40 and CD-86, which are membrane proteins characteristic of the M1-like phenotype.^32^ After a chronic AMF stimulation (*H* = 20 mT, *f* = 97.6 kHz, 3 days) morphology changes, pro-inflammatory cytokines release, and gene expression at the transcriptional level were also evaluated. The expression of M1-like microglia phenotype markers CD40 and CD86 was assessed using flow cytometry across different experimental groups (CTRL, AMF, LMNVs, LMNVs AMF, IFN-γ; the latter as positive control because of the IFN-γ pro-inflammatory activity^33^). As shown in Figures 3c and S6a, just the combined treatment LMNVs + AMF led to a statistically significant upregulation of CD40 expression (25.1 ± 1.7%, *p* < 0.05), a response comparable to that one induced by the IFN-γ treatment (27.4 ± 2.5%, *p* < 0.05). A similar trend was observed for CD86 expression, as shown in Figures 3d and S6b. Notably, in contrast, CD86 expression was already induced by both plain AMF (11.3 ± 0.4%, *p* < 0.05) and LMNV treatment (17.4 ± 0.3%, *p* < 0.05). The most pronounced effect was, however, observed in cells LMNVs + AMF group, where the CD86 expression reached 23.4 ± 0.4% (*p* < 0.05), exceeding the levels induced by IFN-γ (19.2 ± 0.4%, *p* < 0.05).

The morphological changes observed throughout the chronic AMF stimulation qualitatively confirmed a cellular response, as suggested by the confocal images (Figure 4a). Circularity (Figure 4b) and Aspect Ratio (Figure 4c) were quantitatively considered, showing, respectively, a statistically significant reduction and a statistically significant increase in the LMNVs + AMF, and IFN-γ groups. The treatment influenced the actin cytoskeleton, causing an elongation of the microglial cell shape. HMC3 cells treated with LMNVs showed a subtle morphological change compared to CTRL and AMF treatments, with no significant alterations. However, the effect was more pronounced when LMNVs were combined with AMF stimulation, highlighting the role of magneto-thermal activation in modifying cellular structure. Of particular interest, the morphology of microglia treated with LMNVs + AMF is closely resembling that one of IFN-γ-stimulated cells.^32^ These results suggest that the observed morphological alterations may be indicative of functional changes associated with M1-like activation, further highlighting the potential of the proposed approach in modulating microglial behavior.

**Figure 4:**
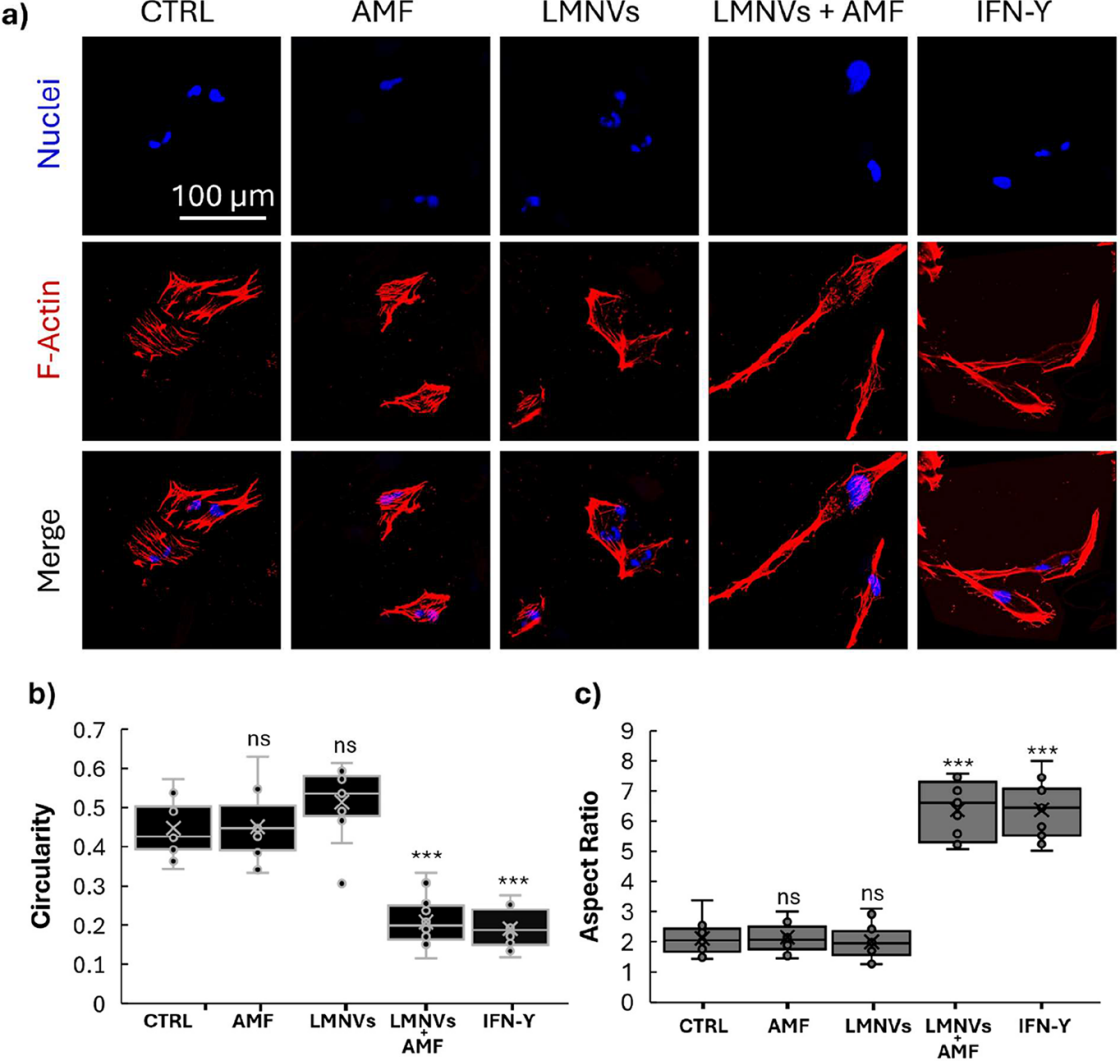
Morphological analysis of microglia after chronic stimulation. (a) Representative confocal images of microglia cells after the stimulation; quantitative evaluation of circularity (b) and aspect ratio (c) as parameters to underline the changes in morphology upon different treatments (ns *p* > 0.05, *** *p* < 0.001).

qRT-PCR analysis was conducted to further characterize the microglial response to the LMNVs + AMF treatment, by evaluating the transcription levels of key pro-inflammatory (M1-like) marker genes; ELISA was conducted to evaluate the corresponding cytokine release into the culture supernatant.

The analyzed genes included IL-6 (Figure 5a), IL-8 (Figure 5b), and TNF-α (Figure 5c), which are commonly associated with the M1-like microglial phenotype. Their mRNA transcription was assessed under all the previously introduced experimental conditions. The obtained results indicate that AMF alone did not significantly alter cytokine-related gene transcription, suggesting that the magnetic field in the absence of nanoparticles is not sufficient to activate microglia. Similar considerations can be applied to the treatment with plain LMNVs. Conversely, as reported in Figure 5a,b, the LMNVs + AMF treatment induced a significant increment of IL-6 and IL-8 transcription respectively by 2.5 ± 0.2 fold and 3.2 ± 0.2 fold, reaching levels comparable to those induced by IFN-γ (2.9 ± 0.2 fold and 3.5 ± 0.2 fold). TNF-α transcription followed a similar trend, though the increase was slightly less pronounced with respect to IL-6 and IL-8, with 1.5 ± 0.1 fold for LMNVs + AMF and 1.7 ± 0.1 fold for IFN-γ (Figure 5c).

**Figure 5:**
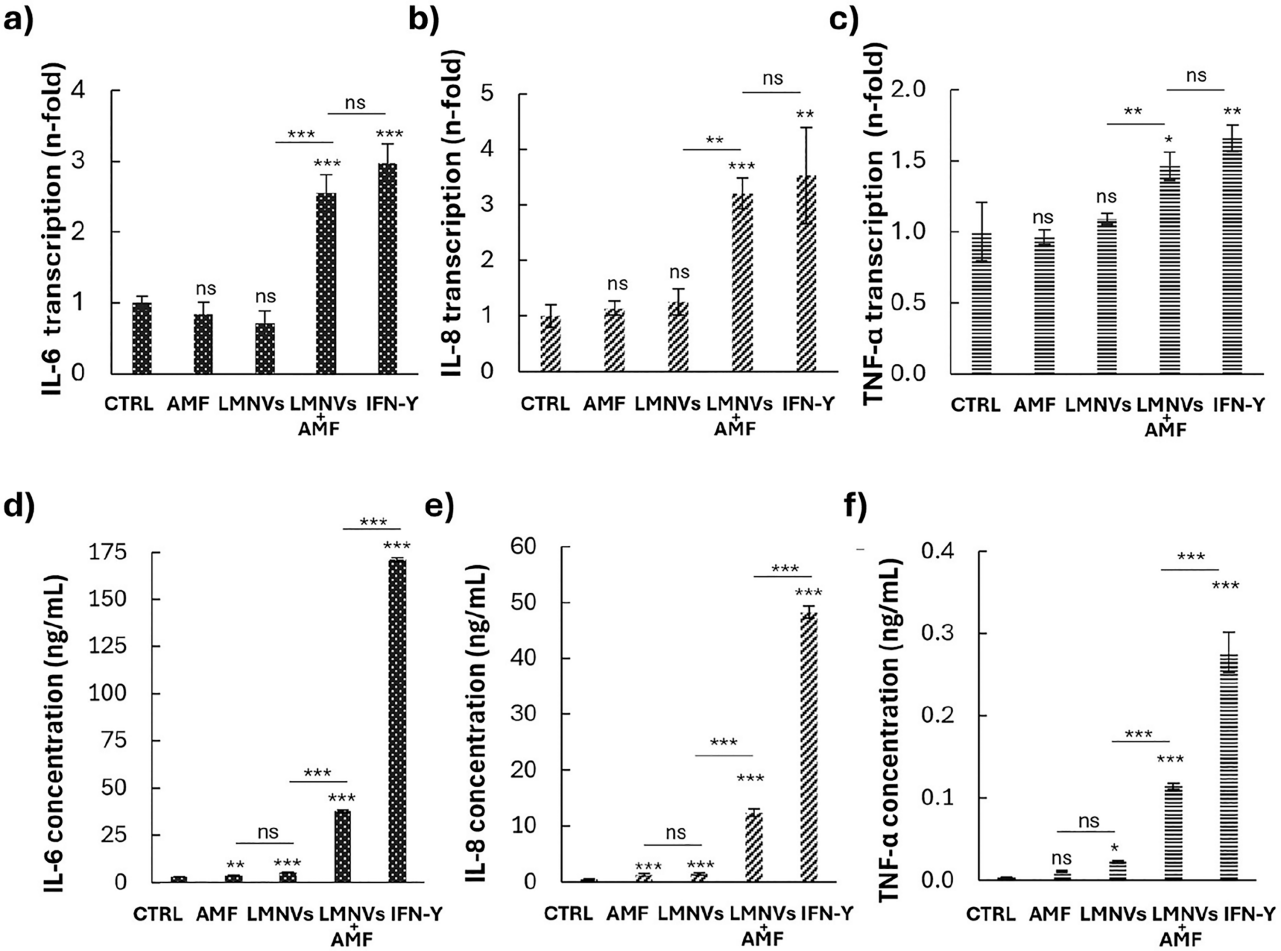
Gene transcription and cytokines release. Relative mRNA quantification of IL-6 (a), IL-8 (b), and TNF-α (c) upon all the experimental treatments; corresponding ELISA results to quantify the secretion of IL-6 (d), IL-8 (e), and TNF-α (f) in the culture supernatant (ns *p* > 0.5, * *p* < 0.05, ** *p* < 0.01, *** *p* < 0.001).

To further validate the transcriptional data at the protein level, ELISA were conducted to quantify the secretion and release of IL-6, IL-8, and TNF-α in the culture supernatants. The results confirmed the trends observed in gene transcription. The single treatments with AMF or LMNVs induced a moderate but significant increase in IL-6, IL-8, and TNF-α release; however, when LMNVs were combined with AMF stimulation, the levels of cytokines increased significantly, confirming that the combined treatment is highly effective in driving microglial activation (Figure 5d–f). The observed slight discrepancies between qRT-PCR and ELISA results may be related to the distinction between mRNA transcription and protein synthesis; while qRT-PCR measures mRNA levels at a specific time, post-transcriptional regulation, including mRNA stability, translational efficiency, and protein degradation, can cause variations in cytokine levels that are independent of gene transcription trends.^34^ In particular, the increase in IL-6 and IL-8 secretion detected by ELISA following AMF or LMNV treatments alone, despite no significant changes in their mRNA expression compared to the control group, suggests the involvement of post-transcriptional mechanisms.^35^ These cytokines may be stored intracellularly and released upon stimulation without requiring new mRNA synthesis. Additionally, enhanced mRNA stability or increased translational efficiency could contribute to higher protein accumulation over time, even in the absence of detectable transcriptional upregulation at the measured time points. TNF-α exhibited high secretion levels in ELISA despite only a modest increase in mRNA expression. This can be attributed to its rapid and tightly regulated production: TNF-α mRNA typically has a short half-life, allowing for rapid translation upon stimulation;^35,36^ furthermore, TNF-α secretion is modulated by autocrine and paracrine signaling loops, amplifying its extracellular concentration beyond what is suggested by mRNA expression data.^37^ The secretion kinetics of cytokines also play a crucial role in these discrepancies, as proteins undergo additional processing, including post-translational modifications and transport, before being released into the extracellular environment.^38^

To further explore the molecular mechanisms of the combined effect of LMNVs and AMF on microglia modulation, a transcriptomic profiling was performed. Specifically, four experimental comparisons were analyzed in-depth: AMF vs CTRL, LMNVs vs CTRL, LMNVs + AMF vs CTRL, and IFN-γ vs CTRL. From these comparisons, the significantly differentially expressed genes (DEGs) were identified, including the number of upregulated and downregulated genes, as summarized in Table S1. The data analysis (volcano plots reported in Figure 6) revealed both common and condition-specific transcriptional mechanisms that could explain the maintenance of microglial homeostasis, as well as the induction of M1-like microglia phenotype. Notably, we identified two main subsets of commonly modulated genes: one shared between the AMF and LMNVs groups, largely reflecting homeostatic regulation, and another shared between the IFN-γ and LMNVs + AMF groups, enriched in pro-inflammatory and immunogenic pathways.

**Figure 6:**
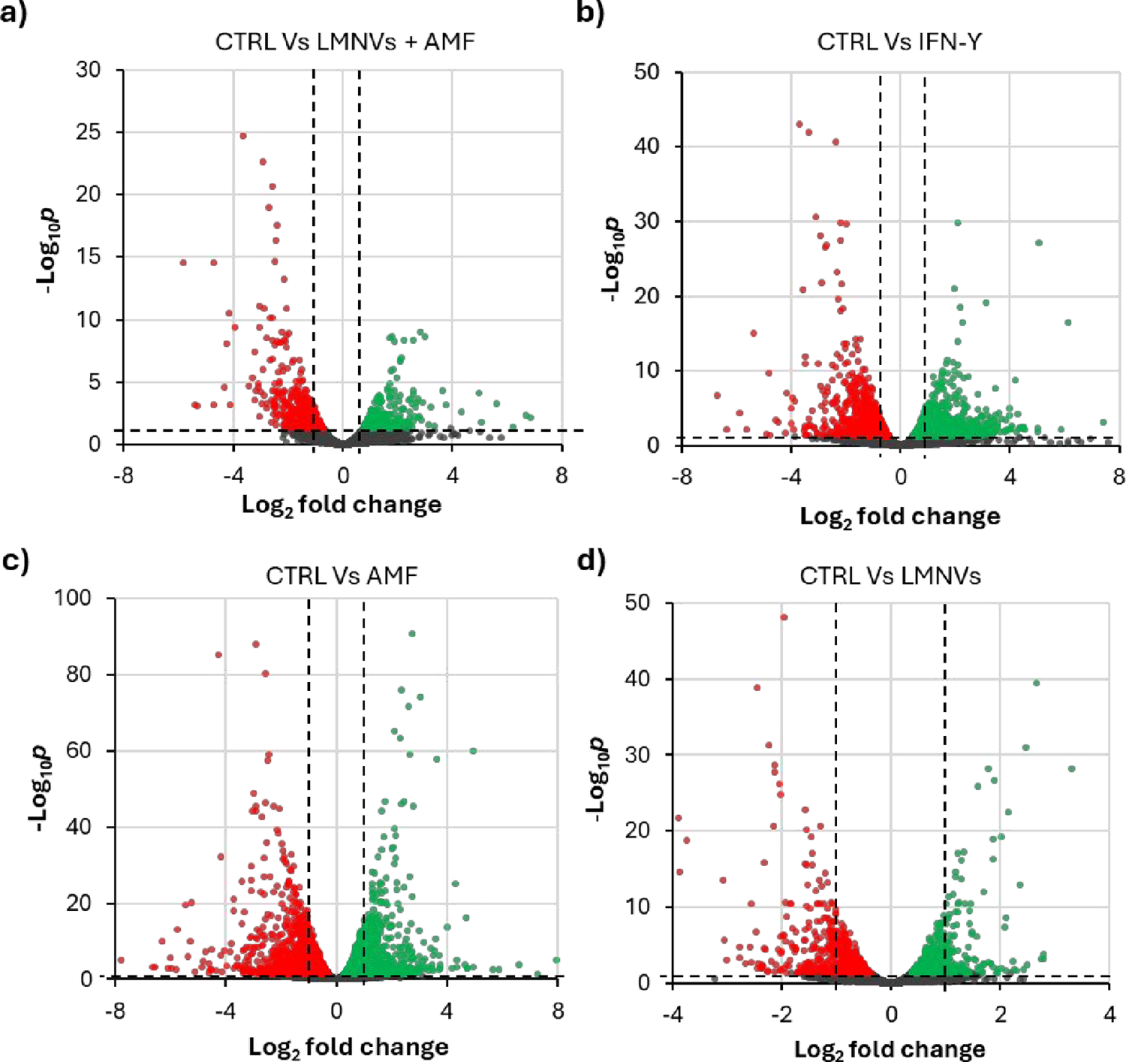
Vulcano plots reporting the transcriptomics data. The plots report the comparison between (a) LMNVs + AMF vs CTRL, (b) IFN-γ vs CTRL, (c) AMF vs CTRL, and (d) LMNVs vs CTRL.

In addition, a small core of genes, including TNF and NFKBIA, was consistently expressed across all experimental conditions, suggesting the presence of a basal NF-κB/TNF activity that is maintained independently from external stimuli. Microglia exposed to either AMF or LMNVs revealed a transcriptional pattern enriched in genes associated with homeostatic regulation and anti-inflammatory restraint. Among DEGs, ITGB8 and RCAN1 stood out as key regulators, and they were upregulated only in the comparisons AMF vs CTRL and LMNVs vs CTRL. ITGB8 encodes the β8 subunit of the α_V_β_8_ integrin, a receptor complex essential for the activation of latent TGF-β in the CNS. Active TGF-β is a key factor in microglial identity, maintaining these cells in a surveillant and noninflammatory state while suppressing NF-κB-driven transcription of pro-inflammatory cytokines such as TNF and IL-6.^39^ Conditional deletion of ITGB8 in the CNS reduces TGF-β activation and results in microglia adopting a reactive transcriptome closely resembling that of TGF-β1-deficient cells, underscoring the critical role of αVβ8–TGF-β signaling in sustaining microglial homeostasis.^40^ The upregulation of ITGB8 in AMF and LMNVs groups likely reflects an enhanced capacity to activate TGF-β and maintain suppression of NF-κB/TNF signaling.^41^ A similar role can be attributed to the significant upregulation of RCAN1, which emerged as another gene consistently expressed under noninflammatory conditions. RCAN1 is an established inhibitor of calcineurin-NFAT signaling and has also been shown to attenuate NF-κB activity.^42,43^ In macrophages and monocytes, RCAN1 deficiency results in exaggerated NF-κB activation and enhanced production of pro-inflammatory mediators, while its induction acts as a negative regulator of cytokine release.^43^ In this case, RCAN1 expression may thus serve as a safeguard against inappropriate inflammatory activation, reinforcing the homeostatic phenotype and attenuating NF-κB pathway.^42^

The upregulation of TNF, as well as NFKBIA, does not necessarily indicate inflammation, as basal TNF expression in microglia is a well-established physiological mechanism contributing to synaptic scaling and neuronal plasticity.^44,45^ Likewise, NFKBIA encodes IκBα, the canonical negative feedback regulator of NF-κB. Its induction reflects a poised but restrained state, in which NF-κB activity is counterbalanced by inhibitory mechanisms.^46^ The coexpression of TNF and NFKBIA across all experimental classes, therefore, indicates a basal state of readiness, kept in check by the combined action of ITGB8 and RCAN1.

Conversely, in IFN-γ-treated microglia and in cells exposed to LMNVs combined with AMF (LMNVs + AMF), this balance was overridden. Within these experimental groups, the upregulation of CD14, SOX9, HMGCS1/HMGCR, TRPM2, and KCNN1 was observed, all of which strongly associated with microglial inflammatory activation. CD14 functions as a coreceptor for TLR4, enhancing the sensitivity of microglia and amplifying NF-κB-mediated transcription of pro-inflammatory cytokines. The CD14 upregulation typically characterizes microglia in a pro-inflammatory state and enhancing the production of cytokines such as TNF, IL-6, and IL-1β. Reed-Geaghan et al., in their in vivo study, demonstrate that without CD14 microglia exhibit an attenuated inflammatory response typical of M1.^47^ SOX9, typically linked to astrocytic differentiation, has also been implicated in inflammatory glial responses and may reflect astrocyte–microglia crosstalk during neuroinflammation.^48^ The mevalonate pathway genes HMGCS1 and HMGCR, although central to cholesterol biosynthesis, also drive metabolic reprogramming that enhances innate immune training and promotes NF-κB and TLR signaling, processes that are suppressed by statin treatment in macrophages and microglia.^49^ The upregulation of TRPM2 and KCNN1 provides insights into the sustained Ca^2^^+^ dynamics observed following LMNV-assisted magneto-thermal stimulation. TRPM2, a Ca^2^^+^-permeable, nonselective cation channel that acts as a key sensor of oxidative stress and thermal stimuli, has been directly linked to TNF and IL-6 release from microglia, and its activity contributes to pathological inflammation.^50^ The upregulation of KCNN1 encodes the small-conductance Ca^2^^+^-activated K^+^ channel SK1; in particular, SK channels, activated by Ca^2^^+^/calmodulin, contribute to membrane hyperpolarization during Ca^2^^+^ entry.^51^ The selective induction of KCNN1 under inflammatory conditions suggests a synergistic mechanism with TRPM2: Ca^2^^+^ influx through TRPM2 activates SK1, which in turn hyperpolarizes the membrane, favoring further Ca^2^^+^ entry through TRPM2 and other channels. This establishes a positive feedback loop sustaining Ca^2^^+^ signaling, crucial for maintaining M1 polarization.^51^

We would like to stress that, while in this study we referred to the classical M1/M2 framework to describe microglial activation, this binary classification provides a simplified representation of the complex and dynamic spectrum of microglial phenotypes observed in GBM. Recent single-cell and spatial transcriptomic analyses have revealed that microglia and tumor-associated macrophages display heterogeneous and overlapping functional states, coexpressing genes typically associated with both pro and anti-inflammatory programs.^4^ Therefore, the M1/M2 terminology used here should be regarded as a conceptual simplification adopted to describe dominant polarization trends, rather than a definitive taxonomy of microglial activation. Our findings should thus be interpreted within a broader multidimensional framework of microglial functional reprogramming in the GBM microenvironment.

Consistently, Ca^2^^+^ imaging experiments showed that LMNVs + AMF stimulation elicited a continuous rise in intracellular Ca^2^^+^, which was abolished under Ca^2^^+^-free extracellular conditions, while only transient responses were detected in AMF controls, likely reflecting release from the endoplasmic reticulum store. These results, in the LMNVs+AMF and IFN-γ experimental groups, support extracellular influx as indispensable for prolonged signaling, with TRPM2 and KCNN1 as key molecular mediators. In addition to these pro-inflammatory regulators, IFN-γ and LMNVs + AMF conditions also showed increased expression of genes potentially associated with ICD, notably HSPA2 and MICA. HSPA2, a member of the HSP70 family, acts as a danger-associated molecular pattern when exposed on the cell surface, stimulating dendritic cell activation and antigen presentation.^52^ HSPA2 can also be linked to the increase in temperature related to the combination of LMNVs + AMF; MICA, a ligand for the activating receptor NKG2D on NK and CD8^+^ T cells, instead enhances immune recognition of stressed or transformed cells.^53^ Their upregulation indicates that activated microglia under IFN-γ or LMNVs + AMF stimulation not only adopt a classical pro-inflammatory phenotype, but also gain immunogenic features that could contribute to antitumor responses.

In line with the observed elongated morphology of microglia under IFN-γ and LMNVs + AMF treatments, the transcriptomic analysis revealed a significant upregulation of FN1 (fibronectin) and DBN1 (drebrin 1). FN1, a key extracellular matrix component, enhances microglial adhesion, phagocytic activity, and cytoskeletal reorganization, features closely associated with cellular elongation and activation states.^54^ DBN, an actin-binding protein, stabilizes F-actin by altering filament architecture, links actin to microtubules, and supports elongation.^55^

Collectively, obtained data support the hypothesis that LMNVs act as efficient nanostransducers, and that their activation through AMF stimulation significantly promote a pro-inflammatory effect. The substantial upregulation of both cytokine gene transcription and secretion suggests that this approach effectively shifts microglia toward a pro-inflammatory phenotype.

### 3.4 Magneto-Thermally Activated Microglia against GBM Cells

Chronic activation of microglia is linked to inflammatory responses that can influence the progression of neurodegenerative diseases and brain tumors.^1,56^ The M1-like microglial phenotype has pro-inflammatory effects, and plays a crucial role in antitumor immune responses by releasing cytokines that can inhibit tumor growth.^57^ Based on these considerations, we investigated whether magneto-thermally activated microglia could suppress the viability and proliferation of both immortalized glioblastoma cells (U87-MG) and patient-derived cells. All experimental groups were incubated with the corresponding microglia-conditioned medium for 4 days. Subsequently, LIVE/DEAD, Qant-iT PicoGreen assay, and WST-1 assay were performed. The LIVE/DEAD assay results revealed a significant reduction in cell viability in patient-derived glioblastoma cells exposed to microglia-conditioned medium. As shown in Figure 7a,b, GBM cells treated with conditioned medium from microglia stimulated with LMNVs + AMF exhibited an even higher percentage of cell death (49.3 ± 2.6%) compared to those treated with IFN-γ-conditioned medium (44.5 ± 7.5%). Conversely, no significant differences in cell viability were observed in cells exposed to conditioned medium from microglia treated with either LMNVs or AMF alone, compared to the untreated control (CTRL). These results were further corroborated by proliferation and metabolic activity assays. Qant-iT PicoGreen analysis demonstrated a substantial reduction in cell proliferation, with decrements to 68.6 ± 1.8 and 56.2 ± 2.3% in glioblastoma cells treated with LMNVs + AMF-conditioned medium and IFN-γ-conditioned medium, respectively (Figure 8a). Additionally, a significant reduction in metabolic activity was observed, with a decrement to 27.8 ± 3.5% in patient-derived GBM cells incubated with LMNVs + AMF-conditioned medium and to 51.3 ± 1.6% for those incubated in the IFN-γ conditioned medium (Figure 8b). Immunostaining for the proliferation marker *K*_i_-67 further substantiated these results, revealing a significant decrease in the percentage of *K*_i_-67^+^ nuclei in patient-derived GBM cells treated with conditioned medium from LMNVs + AMF-stimulated microglia, with reductions in proliferation rates to 44.0 ± 6.9% (Figure 9a,b).

**Figure 7:**
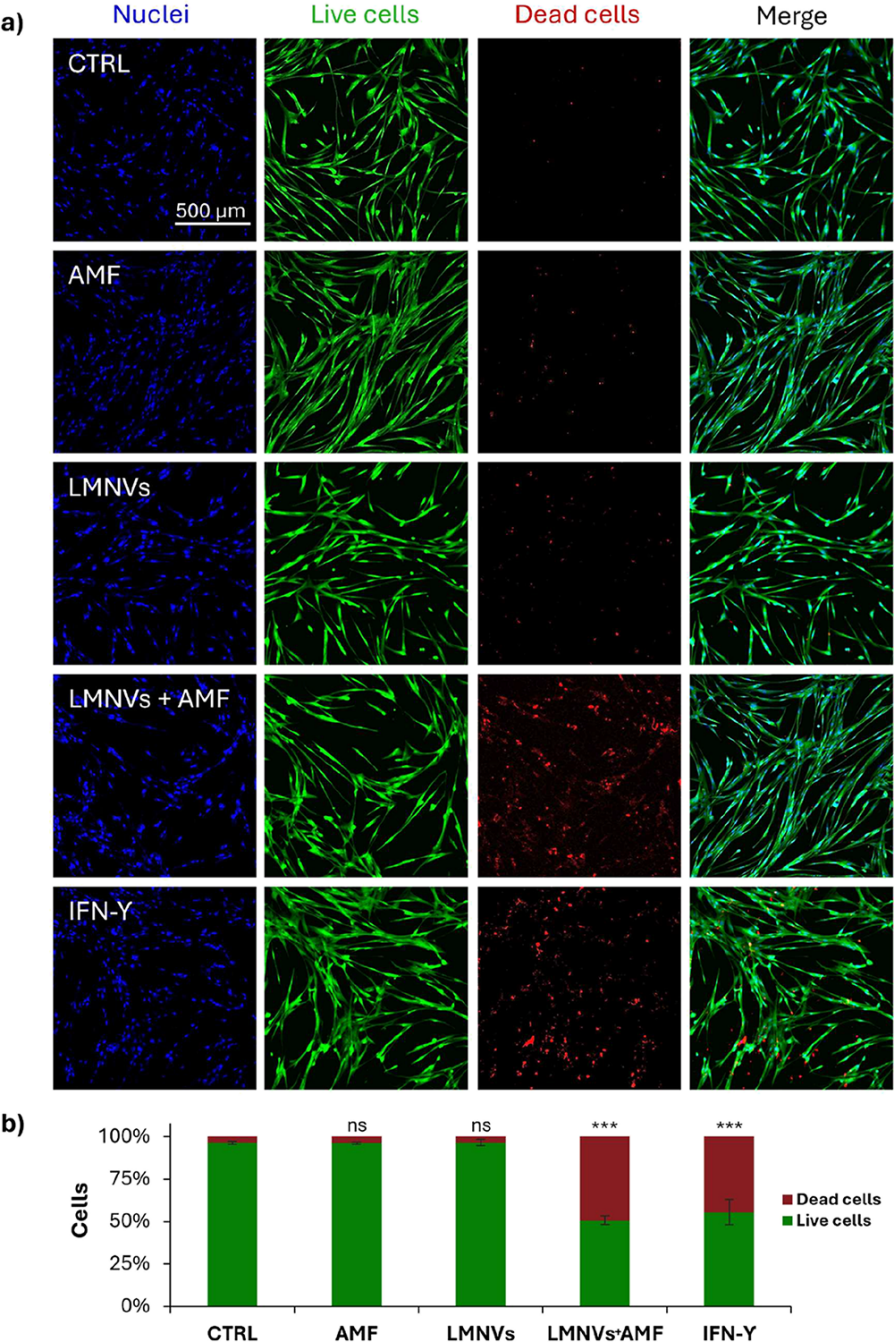
Cell viability in patient-derived GBM cultures after microglia-conditioned medium treatment. (a) Representative confocal images and (b) quantitative analysis (ns *p* > 0.5, *** *p* < 0.001).

**Figure 8:**
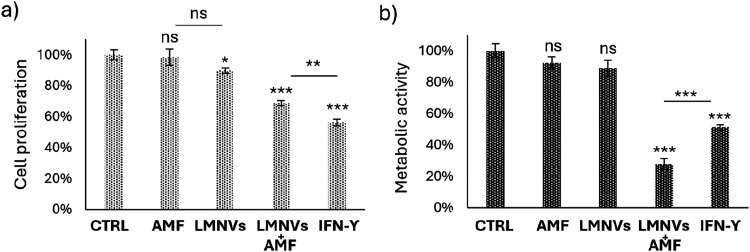
Patient-derived GBM cells exposed to microglia-conditioned media. Cell proliferation (a) and metabolic activity (b) for the different experimental groups (ns *p* > 0.5, *** *p* < 0.001).

**Figure 9:**
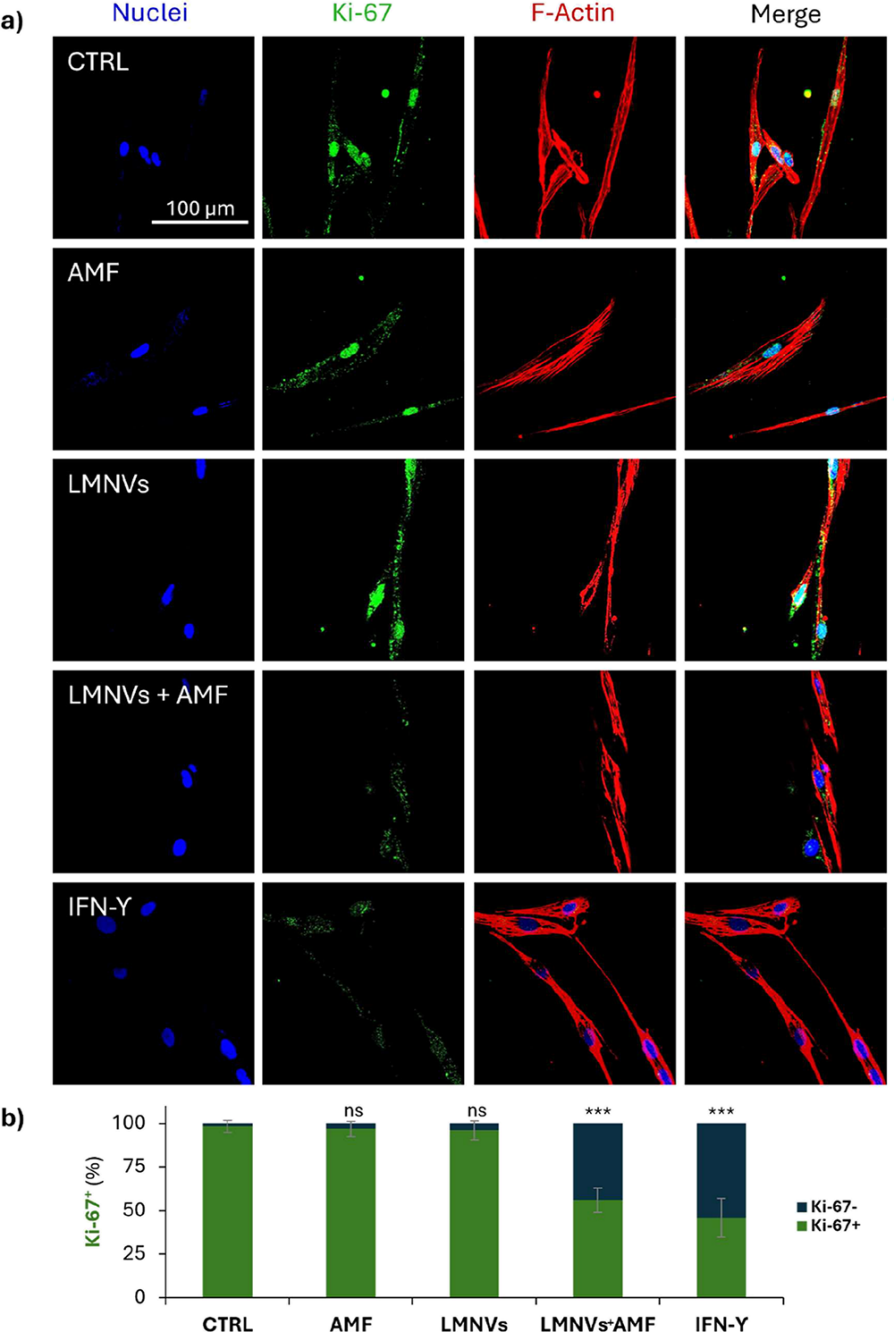
Cell proliferation activity in patient-derived GBM cultures after microglia-conditioned medium treatment. (a) Representative confocal images and (b) quantitative analysis of *K*_i_-67 expression (ns *p* > 0.5, *** *p* < 0.001).

Comparable trends were also observed in immortalized U87-MG cultures, concerning viability (Figure S7a,b), *K*_i_-67 expression (Figure S8a,b), proliferation (Figure S9a), and metabolic activity (Figure S9b).

### 3.5 Immunogenic Cell Death in GBM Cells

The damage-associated molecular hallmarks related to HMGB1 translocation and CRT expression were examined to determine whether GBM cells exhibited features consistent with ICD in both U87-MG and GBM patient-derived cells^58,59^ following exposure to microglia-conditioned media.

Immunofluorescence staining revealed that HMGB1 was predominantly nuclear across all experimental conditions (Figure 10a). However, a statistically significant cytoplasmic expression of HMGB1 was observed in both LMNVs + AMF and IFN-γ groups in comparison to the control (*p* < 0.001), with a percentage of cells of 92.3 ± 13.1 and 92.3 ± 7.4%, respectively (Figure 10b). The translocation of HMGB1 within the cytoplasm is indicative of its eventual release into the extracellular space, a recognized late-stage event in ICD. The similarity between LMNVs + AMF and IFN-γ suggests a comparable level of HMGB1 mobilization and a translocation from the nucleus to the cell membrane, pointing to immunogenic stress induction.^60^ Similar behavior has been observed for U87-MG cells (Figure S10a), with a cell percentage with an expression of HMGB1 in the cytoplasm of 97.6 ± 1.6% in LMNVs + AMF and of 96.8 ± 1.4% in IFN-γ (3.3 ± 2.7% in control, *p* < 0.001; Figure S10b).

**Figure 10:**
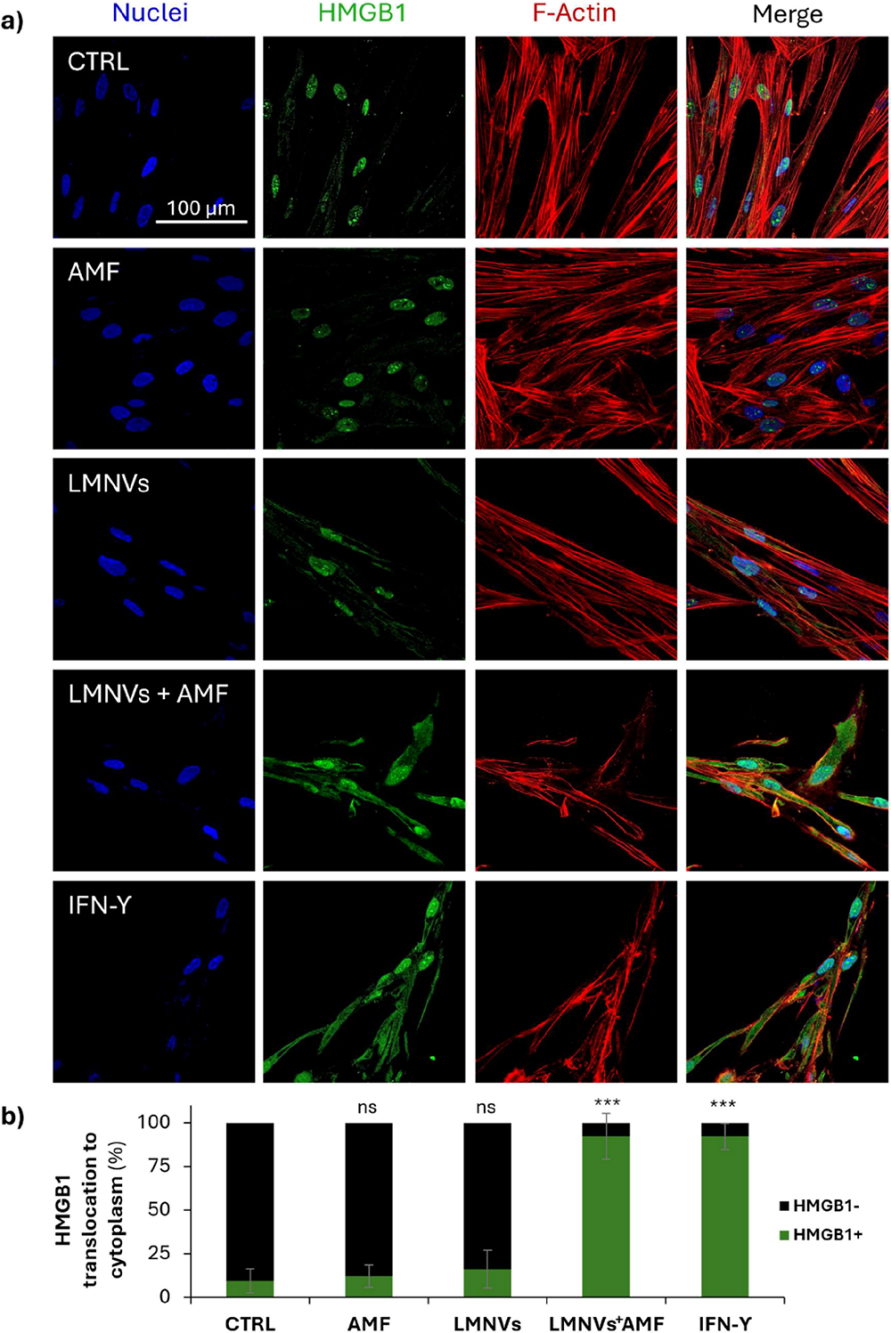
Immunostaining for HMGB1, a marker related to immunogenic cell death (ICD), after the treatment of patient-derived GBM cells with microglia-conditioned medium. (a) Representative confocal images and (b) quantitative analysis considering the HMGB1 translocation from nuclei to cytoplasm (ns *p* > 0.05, *** *p* < 0.001).

Although CRT is physiologically localized in the endoplasmic reticulum (ER), low-level signal can often be detected in the cytoplasm even under nonstressed conditions due to its dynamic shuttling among compartments or basal expression variability, as shown in CTRL, AMF, and LMNVs groups, especially for GBM-patient-derived cells (Figure 11a,b). Conversely, both LMNVs + AMF and IFN-γ induced a statistically significant increase in the cytoplasm (*p* < 0.001 with respect to the control), with a percentage of cells positive to CRT, respectively, of 94.6 ± 6.8 and 91.2 ± 8.6%, reflecting ER stress-induced translocation. This translocation acts as an “eat-me” signal for dendritic cells and is a key early marker of ICD.^61^ Similar data have been observed for U87-MG cells, despite a lower signal (Figure S11a), with a percentage of cells positive to CRT of 96.3 ± 3.1% in LMNVs + AMF and of 93.0 ± 3.2% in IFN-γ (2.7 ± 1.9% in control, CRTL (*p* < 0.001; Figure S11b).

**Figure 11:**
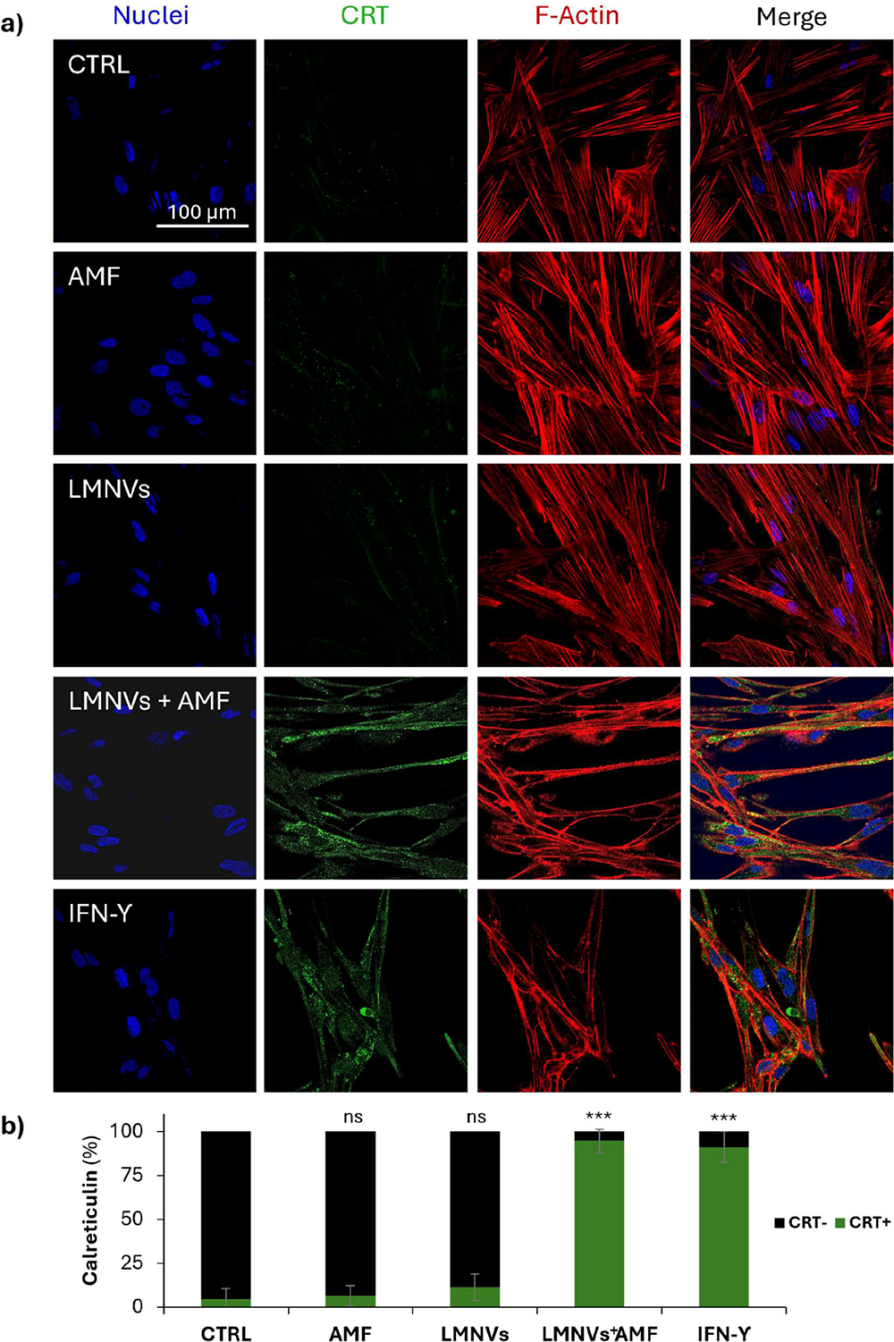
Immunostaining for calreticulin (CRT), a marker related to immunogenic cell death (ICD), after the treatment of patient-derived GBM cells with microglia-conditioned medium. (a) Representative confocal images and (b) quantitative analysis (ns *p* > 0.05, *** *p* < 0.001).

The concurrent observation of HMGB1 translocation and CRT cytoplasmic accumulation strongly support that GBM cells exposed to LMNVs + AMF undergo immunogenic cell death. CRT externalization precedes cell death and enhances recognition and phagocytosis by antigen-presenting cells, whereas HMGB1 release further matures dendritic cells via toll-like receptors (TLR) interactions. The pattern we observed in LMNVs + AMF mirrors the IFN-γ positive control, strengthening the evidence for ICD induction.^62,63^ According to the ICD cellular pathway, ICD requires the release of multiple damage-associated molecular patterns (DAMPs), including CRT, HMGB1, ATP, and type I interferons, often triggered by ER stress or ROS.^62^ Furthermore, numerous studies confirm that CRT exposure and HMGB1 release alone are insufficient to define bona fide ICD: in vivo validation is still necessary. Nonetheless, our results demonstrate that combining LMNV treatment with AMF stimulation triggers ER stress and the release of DAMPs at levels comparable to IFN-γ, highlighting the potential of this stimulation as a promising ICD inducer in GBM models.

Collected data suggest that the pro-inflammatory environment induced by LMNVs + AMF-stimulated microglia significantly impairs glioblastoma cell viability and proliferation, with effects comparable to those of an IFN-γ treatment. Our findings demonstrate that microglia-derived LMNVs activated by alternating magnetic fields trigger hallmarks of immunogenic cell death, especially in patient-derived GBM cells. The statistically significant CRT translocation and HMGB1 cytoplasmic accumulation suggest robust engagement of ICD pathways, equivalent to the standard IFN-γ positive control. The results confirm the hypothesis that lipid-based magnetic nanomaterials, when stimulated by AMF, can influence the antitumor behavior of microglia with a significant effect even on GBM patient-derived cells, suggesting a promising immunotherapeutic strategy against brain tumors.

## 4 Conclusions

The proposed approach to induce an antitumor microglial phenotype through chronic stimulation with LMNVs + AMF effectively triggers a pro-inflammatory response in microglia. Despite containing an inorganic component, the lipid matrix ensures high biocompatibility, efficient cellular internalization, and excellent stability in aqueous environments under physiological conditions. Furthermore, chronic stimulation induced the expression of specific M1-like markers at levels comparable to those observed in cells treated with IFN-γ, a well-established inflammatory promoter. These findings have been further validated by analyzing the secretion of inflammatory cytokines and their relative gene transcription. The transcriptomic profiling further supported and expanded the observations, revealing the coexistence of homeostatic and pro-inflammatory programs depending on the experimental condition. Specifically, AMF and LMNVs alone promoted the regulation of key genes involved in maintaining a noninflammatory microglial state (ITGB8, RCAN1), whereas the combined LMNVs + AMF treatment induced a clear shift toward an M1-like phenotype. This was marked by the upregulation of pro-inflammatory mediators (CD14, SOX9, TRPM2, KCNN1) and the induction of immunogenic signals (HSPA2, MICA) in both LMNVs + AMF and IFN-γ groups. The microglia modulation was also consistent with the observed morphological elongation, supported by the expression of cytoskeleton-related genes (FN1, DBN1). Notably, the cytokine-enriched conditioned medium had a significant cytotoxic effect on both immortalized and patient-derived GBM cells, leading to reduced cell viability, metabolic activity, and proliferation. Consistently, the expression of DAMPs, such as HMGB1 and CRT, was also detected, further supporting the occurrence of immunogenic cell death in both cell lines, particularly in the patient-derived GBM cells.

Our in vitro observations align with recent in vivo findings by Jeon et al.^64^ who demonstrated that localized mild magnetic hyperthermia, achieved through superparamagnetic nanoparticles and AMF, can activate microglia in the hippocampus of mice in a thermal dose-dependent manner. This activation was associated with increased expression of HSP70 and the autophagy marker LC3II, indicating a functional reprogramming toward a pro-inflammatory and potentially phagocytic state, consistent with the response we observed following the LMNVs + AMF treatment.

To the best of our knowledge, this is the first example of a nanomaterial, combined with an externally applied magnetic stimulus under clinically relevant parameters, capable of inducing an antitumor microglial phenotype with effects on glioblastoma cells. While the results reported by Jeon et al.^64^ underscore the ability of stimulus-responsive nanomaterials to modulate microglial activity through nonpharmacological means, it would be of particular interest to investigate whether similar effects could be replicated in vivo within the context of a tumor-bearing model to directly assess the therapeutic potential of this strategy against GBM. Given the complexity of immunotherapeutic approaches for GBM, largely due to its heterogeneity, future studies should explore homotypic cell recognition strategies that specifically target patient-derived microglial cells.^65^ One potential approach could involve surface functionalization of the nanoparticles with immune-related proteins from the patients’ cells, both to enhance targeting specificity and facilitate blood-brain barrier (BBB) penetration, which remains a major challenge for CNS therapies.^66^ Although this aspect was not directly evaluated in the present study, a previous work from our group demonstrated that LMNVs^15^ were able to cross a dynamic in vitro BBB model: these findings suggest that the platform is intrinsically capable of BBB crossing, and that functionalization strategies can further enhance this property. Importantly, in the pathological context of GBM, the BBB is often structurally compromised, particularly in the tumor core and peritumoral regions, which can further facilitate LMNVs accumulation. Both uncoated nanovectors and nanovectors camouflaged with patient-derived GBM membranes were tested, and while the coated formulation exhibited superior transport efficiency, even the uncoated version displayed measurable BBB passage.

A potential limitation of our approach lies in the requirement for AMF stimulation, which demands dedicated instrumentation and careful field calibration to avoid unintended heating effects. Nevertheless, AMF-based magnetic hyperthermia has already been clinically investigated for glioblastoma and other solid tumors, with multiple studies confirming its feasibility and safety within well-established biophysical limits.^67^ Importantly, this dependence on AMF can also be viewed as an advantage: it allows remote, noninvasive, and spatially confined activation of LMNVs, thereby minimizing systemic exposure and restricting therapeutic effects to sites of nanoparticle accumulation. Ongoing advances in AMF applicator design, such as image-guided targeting and miniaturized systems, are expected to further enhance the translational potential and the clinical manageability of this approach.

To further validate the efficacy of the proposed approach, future research should employ more complex models, such as organoids or organ-on-chip systems^68^ that integrate both vascular and tumor components, before progressing to preclinical validation in vivo studies.

## Supplementary Material



## Data Availability

All data are available from the authors upon reasonable request.

## Notes

Figure S1: representative TEM image of LMNVs; Figure S2: high-resolution XPS scans for O 1s of IONPs and LMNVs; Figure S3: metabolic activity, proliferation, viability, and LIVE/DEAD assay results on microglia after LMNV incubation; Figure S4: confocal and flow cytometry analyses of LMNV uptake and intracellular colocalization; Figure S5: intracellular calcium dynamics in LMNVs-treated HMC3 cells during acute AMF stimulation; Figure S6: flow cytometry plots of CD40 and CD86 markers expression; Figure S7: LIVE/DEAD assay of U87-MG cells treated with microglia-conditioned medium; Figure S8: immunostaining for *K*_i_-67 of U87-MG cells treated with microglia-conditioned medium; Figure S9: cell proliferation and metabolic activity of U87-MG cells treated with microglia-conditioned medium; Figures S10 and S11: immunostaining for HMGB1 and calreticulin in U87-MG cells; and Table S1: number of differentially expressed genes (DEGs) in each experimental condition (PDF)

## References

[1] Wei J., Gabrusiewicz K., Heimberger A. (2013). The Controversial Role of Microglia
in Malignant Gliomas. Clin Dev Immunol.

[2] Wang G., Zhong K., Wang Z., Zhang Z., Tang X., Tong A., Zhou L. (2022). Tumor-Associated
Microglia and Macrophages
in Glioblastoma: From Basic Insights to Therapeutic Opportunities. Front. Immunol..

[3] Watters J. J., Schartner J. M., Badie B. (2005). Microglia Function in Brain Tumors. J. Neurosci.
Res..

[4] Ransohoff R. M. (2016). A Polarizing
Question: Do M1 and M2Microglia Exist. Nat.
Neurosci..

[5] Feng Q., Xu X., Wei C., Li Y., Wang M., Lv C., Wu J., Dai Y., Han Y., Lesniak M. S., Fan H., Zhang L., Cheng Y. (2021). The Dynamic
Interactions between
Nanoparticles and Macrophages Impact Their Fate in Brain Tumors. Small.

[6] Battaglini M., Marino A., Montorsi M., Carmignani A., Ceccarelli M. C., Ciofani G. (2024). Nanomaterials as Microglia Modulators
in the Treatment of Central Nervous System Disorders. Adv. Healthcare Mater..

[7] Wang J., Lee J. S., Kim D., Zhu L. (2017). Exploration of Zinc
Oxide Nanoparticles as a Multitarget and Multifunctional Anticancer
Nanomedicine. ACS Appl. Mater. Interfaces.

[8] Zhang W., Cao S., Liang S., Tan C. H., Luo B., Xu X., Saw P. E. (2020). Differently Charged Super-Paramagnetic Iron Oxide Nanoparticles
Preferentially Induced M1-Like Phenotype of Macrophages. Front Bioeng. Biotechnol.

[9] Zhang F., Parayath N. N., Ene C. I., Stephan S. B., Koehne A. L., Coon M. E., Holland E. C., Stephan M. T. (2019). Genetic Programming
of Macrophages to Perform Anti-Tumor Functions Using Targeted MRNA
Nanocarriers. Nat. Commun..

[10] Gao X., Li S., Ding F., Liu X., Wu Y., Li J., Feng J., Zhu X., Zhang C. (2021). A Virus-Mimicking Nucleic
Acid Nanogel Reprograms Microglia and Macrophages for Glioblastoma
Therapy. Adv. Mater..

[11] Montorsi M., Pucci C., De Pasquale D., Marino A., Ceccarelli M. C., Mazzuferi M., Bartolucci M., Petretto A., Prato M., Debellis D., De Simoni G., Pugliese G., Labardi M., Ciofani G. (2024). Ultrasound-Activated
Piezoelectric Nanoparticles Trigger
Microglia Activity Against Glioblastoma Cells. Adv. Healthcare Mater..

[12] Bianchi S. (2020). Ultrasound
and Bone: A Pictorial Review. J. Ultrasound.

[13] Riis T. S., Webb T. D., Kubanek J. (2022). Acoustic Properties across the Human
Skull. Ultrasonics.

[14] Cai X., Zhu Q., Zeng Y., Zeng Q., Chen X., Zhan Y. (2019). Manganese
Oxide Nanoparticles As MRI Contrast Agents In Tumor Multimodal Imaging
And Therapy. Int. J. Nanomed..

[15] De
Pasquale D., Pucci C., Desii A., Marino A., Debellis D., Leoncino L., Prato M., Moscato S., Amadio S., Fiaschi P., Prior A., Ciofani G. (2023). A Novel Patient-Personalized
Nanovector Based on Homotypic Recognition and Magnetic Hyperthermia
for an Efficient Treatment of Glioblastoma Multiforme. Adv. Healthcare Mater..

[16] Stuart, B. H. Infrared Spectroscopy: Fundamentals and Applications; Wiley, 2004.

[17] Wildeboer R. R., Southern P., Pankhurst Q. A. (2014). On the Reliable Measurement of Specific
Absorption Rates and Intrinsic Loss Parameters in Magnetic Hyperthermia
Materials. J. Phys. D Appl. Phys..

[18] Wei Y., Han B., Hu X., Lin Y., Wang X., Deng X. (2012). Synthesis
of Fe3O4 Nanoparticles and Their Magnetic Properties. Procedia Eng..

[19] Wilson D., Langell M. A. (2014). XPS Analysis of Oleylamine/Oleic
Acid Capped Fe3O4
Nanoparticles as a Function of Temperature. Appl. Surf. Sci..

[20] Tapeinos C., Marino A., Battaglini M., Migliorin S., Brescia R., Scarpellini A., De Julián Fernández C., Prato M., Drago F., Ciofani G. (2019). Stimuli-Responsive
Lipid-Based Magnetic Nanovectors Increase Apoptosis in Glioblastoma
Cells through Synergic Intracellular Hyperthermia and Chemotherapy. Nanoscale.

[21] Grosvenor A. P., Kobe B. A., Biesinger M. C., McIntyre N. S. (2004). Investigation of
Multiplet Splitting of Fe 2p XPS Spectra and Bonding in Iron Compounds. Surf. Interface Anal..

[22] Biesinger M. C., Payne B. P., Grosvenor A. P., Lau L. W. M., Gerson A. R., Smart R. St. C. (2011). Resolving Surface
Chemical States in XPS Analysis of
First Row Transition Metals, Oxides and Hydroxides: Cr, Mn, Fe, Co
and Ni. Appl. Surf. Sci..

[23] Dutz S., Hergt R. (2013). Magnetic Nanoparticle
Heating and Heat Transfer on a Microscale:
Basic Principles, Realities and Physical Limitations of Hyperthermia
for Tumour Therapy. International Journal of
Hyperthermia.

[24] Herrero
de la Parte B., Rodrigo I., Gutiérrez-Basoa J., Iturrizaga Correcher S., Mar Medina C., Echevarría-Uraga J. J., Garcia J. A., Plazaola F., García-Alonso I. (2022). Proposal of
New Safety Limits for In Vivo Experiments of Magnetic Hyperthermia
Antitumor Therapy. Cancers (Basel).

[25] Pucci C., Degl’Innocenti A., Belenli Gümüş M., Ciofani G. (2022). Superparamagnetic Iron
Oxide Nanoparticles for Magnetic
Hyperthermia: Recent Advancements, Molecular Effects, and Future Directions
in the Omics Era. Biomater Sci..

[26] Marchianò V., Salvador M., Moyano A., Gutiérrez G., Matos M., Yáñez-Vilar S., Piñeiro Y., Rivas J., Martínez-García J. C., Peddis D., Blanco-López M.
C., Rivas M., Ditaranto N., Cioffi N. (2021). Electrodecoration and Characterization
of Superparamagnetic Iron Oxide Nanoparticles with Bioactive Synergistic
Nanocopper: Magnetic Hyperthermia-Induced Ionic Release for Anti-Biofilm
Action. Antibiotics.

[27] Luiz M. T., Dutra J. A. P., Viegas J. S. R., de Araújo J. T. C., Tavares Junior A. G., Chorilli M. (2023). Hybrid Magnetic Lipid-Based
Nanoparticles for Cancer Therapy. Pharmaceutics.

[28] Portilla Y., Mulens-Arias V., Paradela A., Ramos-Fernández A., Pérez-Yagüe S., Morales M. P., Barber D. F. (2022). The Surface
Coating of Iron Oxide Nanoparticles Drives Their Intracellular Trafficking
and Degradation in Endolysosomes Differently Depending on the Cell
Type. Biomaterials.

[29] Brawek B., Garaschuk O. (2013). Microglial
Calcium Signaling in the Adult. Aged and Diseased
Brain. Cell Calcium.

[30] Zhu D., Feng L., Feliu N., Guse A. H., Parak W. J. (2021). Stimulation
of Local Cytosolic Calcium Release by Photothermal Heating for Studying
Intra- and Intercellular Calcium Waves. Adv.
Mater..

[31] Nadezhdin K. D., Neuberger A., Sobolevsky A. I. (2022). Structural Snapshots of the Mechanism
of TRPV2 Channel Activation by Small-Molecule Agonists. Cell Calcium.

[32] Jurga A. M., Paleczna M., Kuter K. Z. (2020). Overview of General
and Discriminating
Markers of Differential Microglia Phenotypes. Front Cell Neurosci.

[33] Lively S., Schlichter L. C. (2018). Microglia Responses to Pro-Inflammatory
Stimuli (LPS,
IFNγ+TNFα) and Reprogramming by Resolving Cytokines (IL-4,
IL-10). Front Cell Neurosci.

[34] Rieckmann J. C., Geiger R., Hornburg D., Wolf T., Kveler K., Jarrossay D., Sallusto F., Shen-Orr S. S., Lanzavecchia A., Mann M., Meissner F. (2017). Social Network Architecture
of Human
Immune Cells Unveiled by Quantitative Proteomics. Nat. Immunol.

[35] Lacy P., Stow J. L. (2011). Cytokine Release from Innate Immune Cells: Association
with Diverse Membrane Trafficking Pathways. Blood.

[36] Vlasova-St
Louis I., Bohjanen P. R. (2017). Post-Transcriptional Regulation of
Cytokine and Growth Factor Signaling in Cancer. Cytokine Growth Factor Rev..

[37] Welser-Alves J. V., Milner R. (2013). Microglia Are the Major
Source of TNF-α and TGF-Β1
in Postnatal Glial Cultures; Regulation by Cytokines, Lipopolysaccharide,
and Vitronectin. Neurochem. Int..

[38] Smith J. A., Das A., Ray S. K., Banik N. L. (2012). Role of Pro-Inflammatory Cytokines
Released from Microglia in Neurodegenerative Diseases. Brain Res. Bull..

[39] Li L., Sun B., Harris O. A., Luo J. (2024). TGF-β Signaling in Microglia:
A Key Regulator of Development, Homeostasis and Reactivity. Biomedicines.

[40] Arnold T. D., Lizama C. O., Cautivo K. M., Santander N., Lin L., Qiu H., Huang E. J., Liu C., Mukouyama Y. S., Reichardt L. F., Zovein A. C., Sheppard D. (2019). Impaired ΑVβ8
and TGFβ Signaling Lead to Microglial Dysmaturation and Neuromotor
Dysfunction. J. Exp. Med..

[41] Bedolla A., Wegman E., Weed M., Stevens M. K., Ware K., Paranjpe A., Alkhimovitch A., Ifergan I., Taranov A., Peter J. D., Gonzalez R. M. S., Robinson J. E., McClain L., Roskin K. M., Greig N. H., Luo Y. (2024). Adult Microglial TGFβ1
Is Required for Microglia Homeostasis via an Autocrine Mechanism to
Maintain Cognitive Function in Mice. Nat. Commun..

[42] Yang X., Yun Y., Wang P., Zhao J., Sun X. (2022). Upregulation of RCAN1.4
by HIF1α Alleviates OGD-Induced Inflammatory Response in Astrocytes. Ann. Clin Transl Neurol.

[43] Pang Z., Junkins R. D., Raudonis R., MacNeil A. J., McCormick C., Cheng Z., Lin T. J. (2018). Regulator
of Calcineurin 1 Differentially
Regulates TLR-Dependent MyD88 and TRIF Signaling Pathways. PLoS One.

[44] Mygind L., Skov-Skov Bergh M., Tejsi V., Vaitheeswaran R., Lambertsen K. L., Finsen B., Metaxas A. (2021). Tumor Necrosis Factor
(Tnf) Is Required for Spatial Learning and Memory in Male Mice under
Physiological, but Not Im-Mune-challenged Conditions. Cells.

[45] Guzmán-Ruíz M. A., Guerrero
Vargas N. N., Ramírez-Carreto R. J., González-Orozco J. C., Torres-Hernández B. A., Valle-Rodríguez M., Guevara-Guzmán R., Chavarría A. (2024). Microglia
in Physiological Conditions and the Importance of Understanding Their
Homeostatic Functions in the Arcuate Nucleus. Front. Immunol..

[46] Yu H., Lin L., Zhang Z., Zhang H., Hu H. (2020). Targeting
NF-ΚB
Pathway for the Therapy of Diseases: Mechanism and Clinical Study. Signal Transduct. Target. Therapy.

[47] Reed-Geaghan E. G., Reed Q. W., Cramer P. E., Landreth G. E. (2010). Deletion
of CD14
Attenuates Alzheimer’s Disease Pathology by Influencing the
Brain’s Inflammatory Milieu. J. Neurosci..

[48] Yu S. S., Li Z. Y., Xu X. Z., Yao F., Luo Y., Liu Y. C., Cheng L., Zheng M. G., Jing J. H. (2022). M1-Type
Microglia Can Induce Astrocytes to Deposit Chondroitin Sulfate Proteoglycan
after Spinal Cord Injury. Neural Regen. Res..

[49] Zhou X., Wu X., Wang R., Han L., Li H., Zhao W. (2024). Mechanisms
of 3-Hydroxyl 3-Methylglutaryl CoA Reductase in Alzheimer’s
Disease. Int. J. Mol. Sci..

[50] Malko P., Syed Mortadza S. A., McWilliam J., Jiang L. H. (2019). TRPM2 Channel in
Microglia as a New Player in Neuroinflammation Associated With a Spectrum
of Central Nervous System Pathologies. Front.
Pharmacol..

[51] Dolga A. M., Culmsee C. (2012). Protective Roles for
Potassium SK/Kca2 Channels in
Microglia and Neurons. Front. Pharmacol..

[52] Dong Y., Li T., Ma Z., Zhou C., Wang X., Li J. (2022). HSPA1A, HSPA2,
and HSPA8 Are Potential Molecular Biomarkers for Prognosis among HSP70
Family in Alzheimer’s Disease. Dis. Markers.

[53] Raulet D. H. (2003). Roles of
the NKG2D Immunoreceptor and Its Ligands. Nat.
Rev. Immunol..

[54] Kim H., Ahn M., Choi S., Kim M., Sim K. B., Kim J., Moon C., Shin T. (2013). Potential
Role of Fibronectin in
Microglia/Macrophage Activation Following Cryoinjury in the Rat Brain:
An Immunohistochemical Study. Brain Res..

[55] Leslie M. (2013). Drebrin Shows
Self-Restraint. J. Cell Biol..

[56] Watters J. J., Schartner J. M., Badie B. (2005). Microglia Function in Brain Tumors. J. Neurosci
Res..

[57] Lanza M., Casili G., Campolo M., Paterniti I., Colarossi C., Mare M., Giuffrida R., Caffo M., Esposito E., Cuzzocrea S. (2021). Immunomodulatory
Effect of Microglia-Released Cytokines in Gliomas. Brain Sci..

[58] Xie D., Wang Q., Wu G. (2022). Research Progress in Inducing Immunogenic
Cell Death of Tumor Cells. Front. Immunol..

[59] Fang K., Yuan S., Zhang X., Zhang J., Sun S. L., Li X. (2025). Regulation of Immunogenic
Cell Death and Potential Applications in
Cancer Therapy. Front. Immunol..

[60] Decraene B., Yang Y., De Smet F., Garg A. D., Agostinis P., De Vleeschouwer S. (2022). Immunogenic
Cell Death and Its Therapeutic or Prognostic
Potential in High-Grade Glioma. Genes Immunity.

[61] Tarr J. M., Young P. J., Morse R., Shaw D. J., Haigh R., Petrov P. G., Johnson S. J., Winyard P. G., Eggleton P. (2010). A Mechanism
of Release of Calreticulin from Cells During Apoptosis. J. Mol. Biol..

[62] Rodrigues M. C., Morais J. A. V., Ganassin R., Oliveira G. R. T., Costa F. C., Morais A. A. C., Silveira A. P., Silva V. C. M., Longo J. P. F., Muehlmann L. A. (2022). An Overview on Immunogenic Cell Death
in Cancer Biology
and Therapy. Pharmaceutics.

[63] Kielbik M., Szulc-Kielbik I., Klink M. (2021). Calreticulin—Multifunctional
Chaperone in Immunogenic Cell Death: Potential Significance as a Prognostic
Biomarker in Ovarian Cancer Patients. Cells.

[64] Jeon B. T., Naveed M., Puleo M., Kim W.-Y., Kim M.-H. (2025). Localized
Brain Stimulation with Mild Magnetic Hyperthermia Promotes Microglia
Activity towards Reactive and Autophagic Phenotypes in Vivo. Sci. Rep.

[65] Li J., Wei Y., Zhang C., Bi R., Qiu Y., Li Y., Hu B. (2023). Cell-Membrane-Coated Nanoparticles for Targeted Drug
Delivery to
the Brain for the Treatment of Neurological Diseases. Pharmaceutics.

[66] Hersh A. M., Alomari S., Tyler B. M. (2022). Crossing
the Blood-Brain Barrier:
Advances in Nanoparticle Technology for Drug Delivery in Neuro-Oncology. Int. J. Mol. Sci..

[67] Rodriguez B., Rivera D., Zhang J. Y., Brown C., Young T., Williams T., Huq S., Mattioli M., Bouras A., Hadjpanayis C. G. (2024). Magnetic
Hyperthermia Therapy for High-Grade Glioma:
A State-of-the-Art Review. Pharmaceuticals.

[68] Thomas G., Rahman R. (2025). Evolution of Preclinical
Models for Glioblastoma Modelling
and Drug Screening. Curr. Oncol. Rep..

