# Supplementary material for: Thermo-Magnetic Induction of Pro-Inflammatory Microglia: A Lipid-Based Nanovector Strategy for Glioblastoma Immunotherapy
Source: ACS Appl Mater Interfaces. 2025 Nov 5;17(46):63253–71. doi: 10.1021/acsami.5c18518 (PMC12635967; doi:10.1021/acsami.5c18518)
Supplement: Supplementary file 1 [file am5c18518_si_001.pdf]

SUPPORTING INFORMATION FOR

Thermo-magnetic induction of pro-inflammatory microglia: A lipid-based nanovector strategy for glioblastoma immunotherapy

Maria Cristina Ceccarelli[1,2], Giuliana Paravizzini[1,3], Attilio Marino[1], Giulia Gigante[1,2], Alessio Carmignani[1] Federico Catalano[4], Mirko Prato[5], Giammarino Pugliese[6], Pietro Fiaschi[7,8], Matteo Battaglini[1*], Gianni Ciofani[1*]*

[1]Istituto Italiano di Tecnologia, Smart Bio-Interfaces, Viale Rinaldo Piaggio 34, 56025 Pontedera, Italy

[2]Scuola Superiore Sant'Anna, The BioRobotics Institute, Viale Rinaldo Piaggio 34, 56025 Pontedera, Italy

[3]Politecnico di Torino, Department of Mechanical and Aerospace Engineering, Corso Duca degli Abruzzi 24, 10129 Torino, Italy

[4]Istituto Italiano di Tecnologia, Electron Microscopy Facility, Via Morego 30, 16163 Genova, Italy

[5]Istituto Italiano di Tecnologia, Materials Characterization Facility, Via Morego 30, 16163 Genova, Italy

[6]Istituto Italiano di Tecnologia, Chemistry Facility, Via Morego 30, Genova, 16163 Italy

[7]IRCCS Ospedale Policlinico San Martino, Department of Neurosurgery, Largo Rossana Benzi 10, 16132, Genova, Italy

[8]University of Genova, Department of Neuroscience, Rehabilitation, Ophthalmology, Genetics, Maternal and Child Health (DiNOGMI), Largo Paolo Daneo 3, 16132, Genova, Italy

*Corresponding Authors: maria.ceccarelli@iit.it; matteo.battaglini@iit.it; gianni.ciofani@iit.it

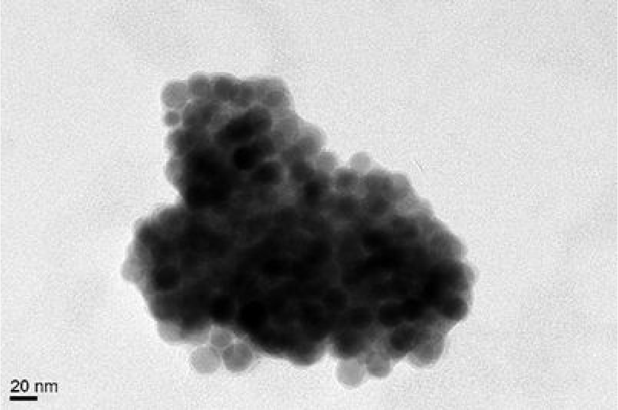

Figure S1. Representative TEM image of LMNVs.

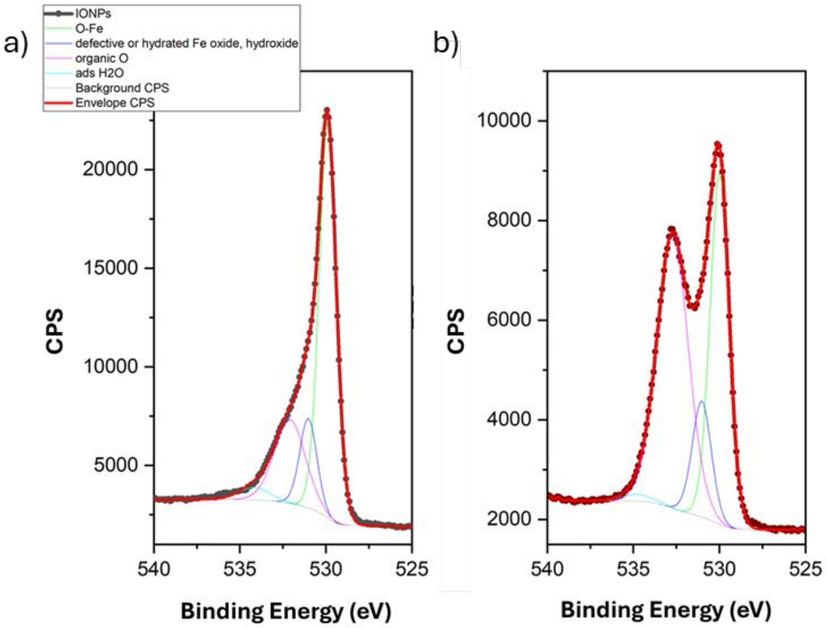

Figure S2. High-resolution XPS scans for O 1s respectively for (a) IONPs and (b) LMNVs.

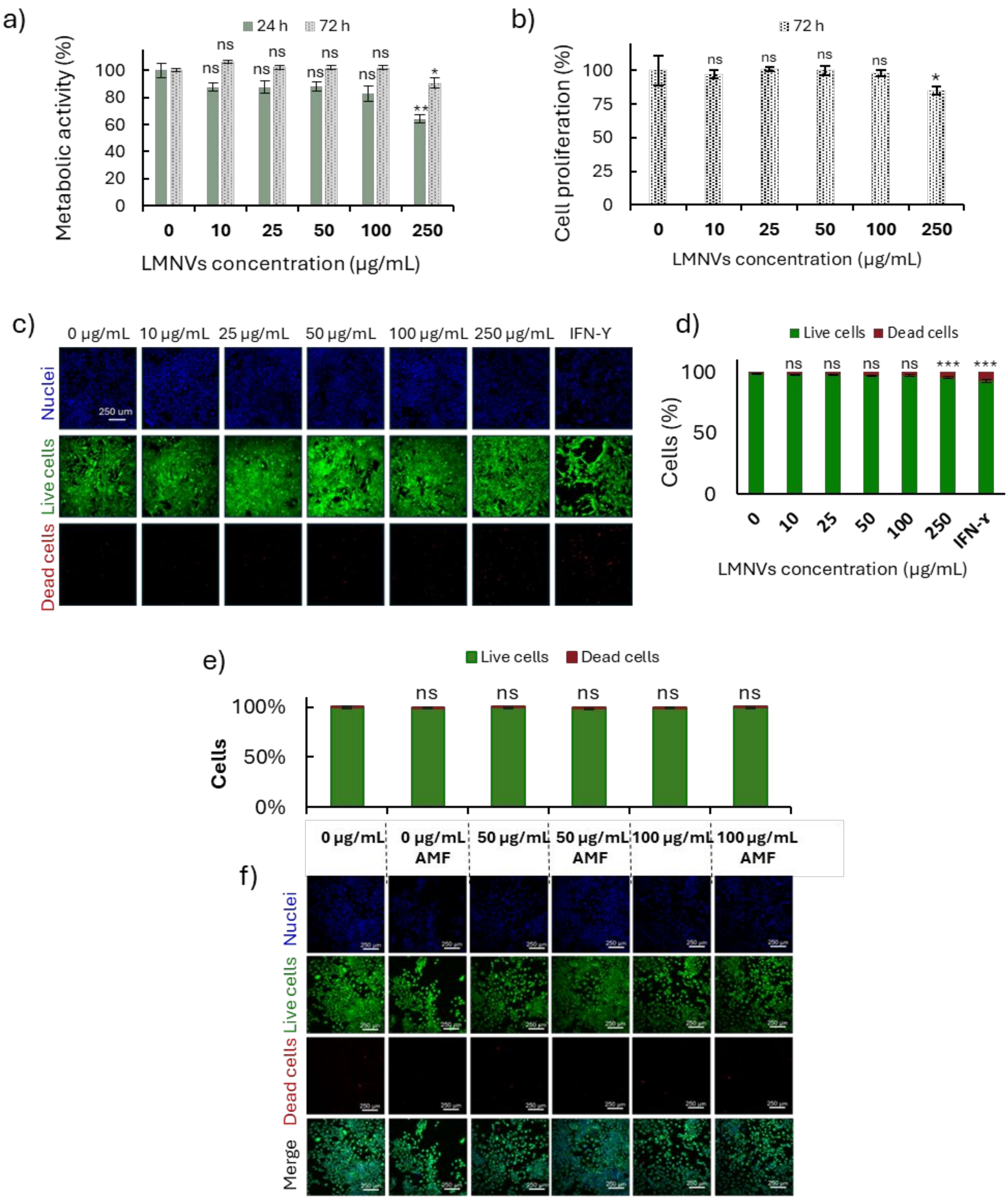

Figure S3. Metabolic activity (a), proliferation (b), and viability (c-d) assessment on microglia following incubation with LMNVs; Quantitative results of LIVE/DEAD assay (e) and representative confocal images (f) to evaluate the most suitable LMNV concentration for AMF stimulation (ns $p > 0.05$, * $p < 0.05$).

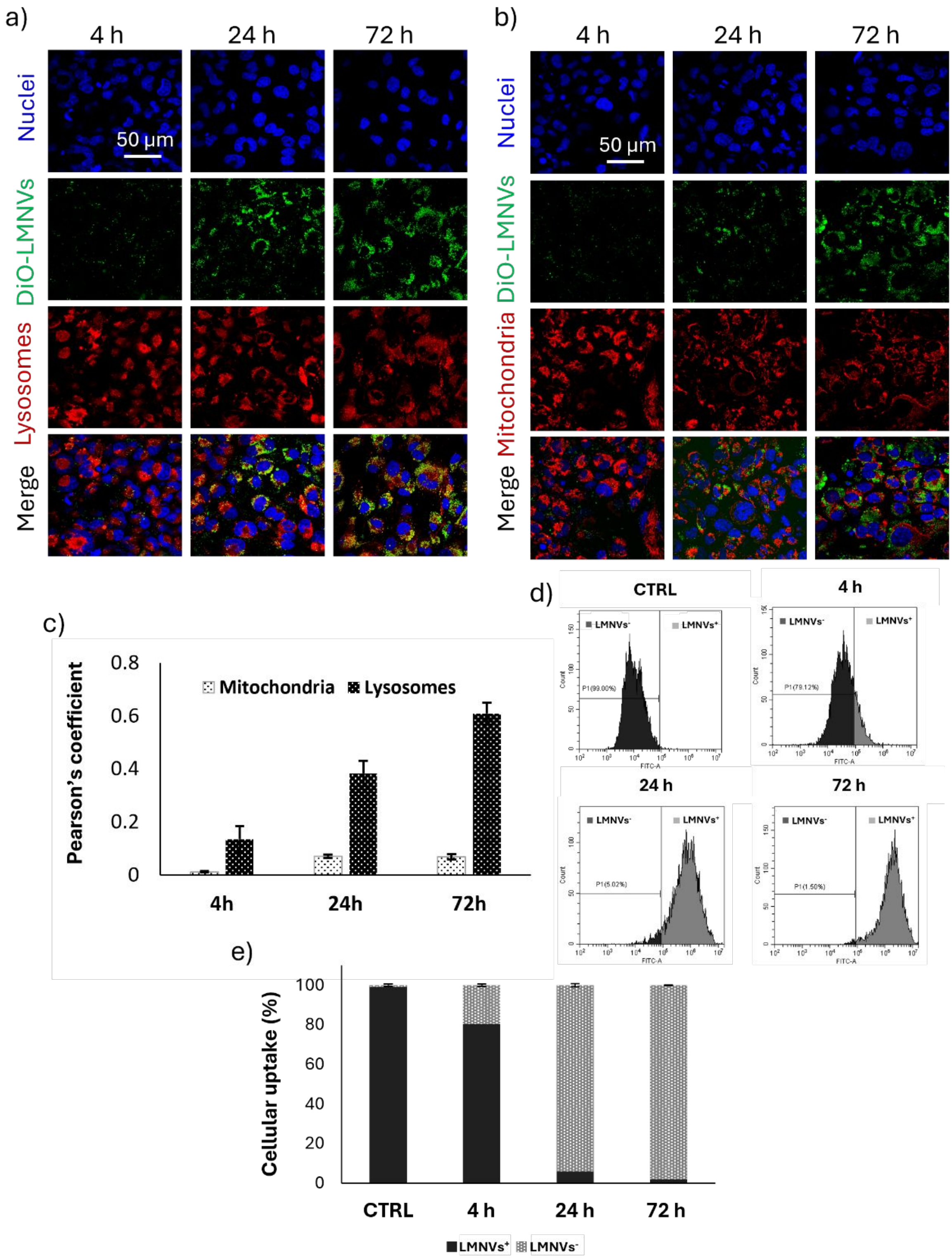

Figure S4. Representative confocal images to visualize the co-localization of Dio-LMNVs (in green) with lysosomes (in red, a) or mitochondria (in red, b) and nuclei (in blue) of microglia cells (HMC3) at 4, 24, and 72 h of LMNV incubation. (c) Quantitative evaluation of co-localization through Pearson's coefficient to perform the overlap analysis. (d) Representative flow cytometry plots of fluorescence levels of cells after LMNV treatment, indicative of nanoparticle internalization. (e) Quantitative data of cellular uptake analyzed through flow cytometry.

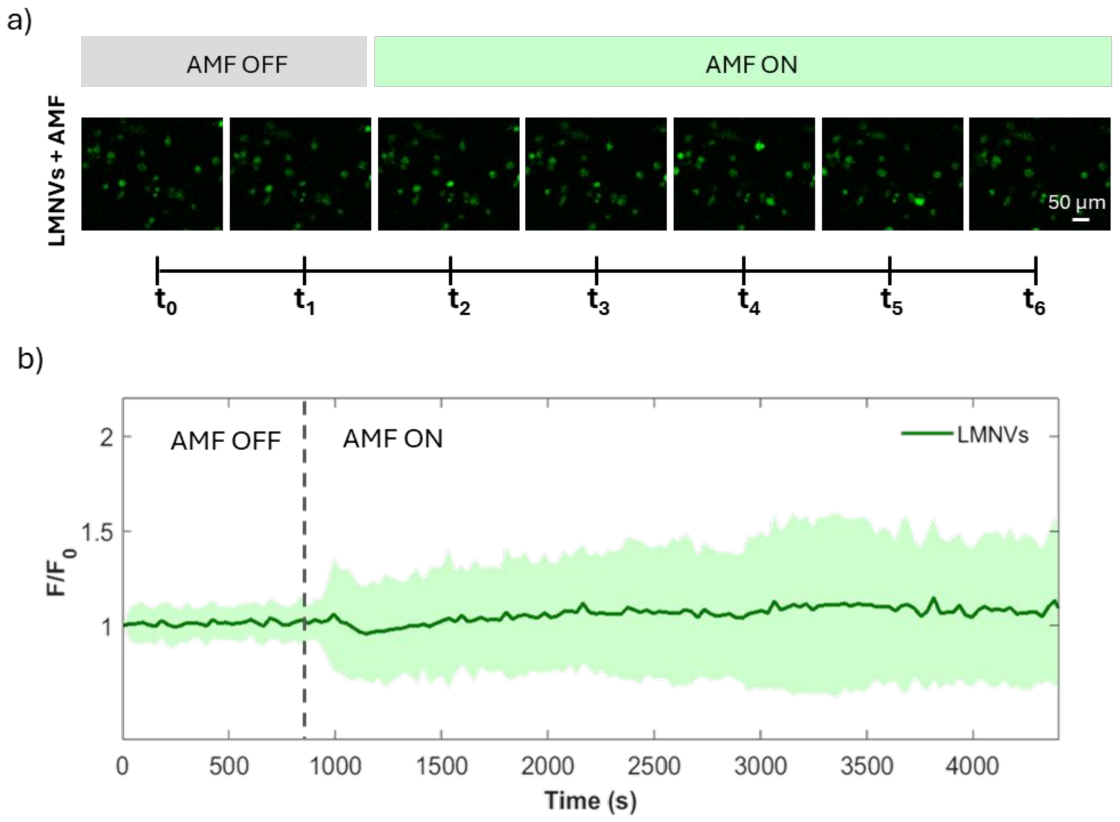

Figure S5. (a) Representative time-lapse confocal images showing intracellular calcium dynamics in LMNV-treated HMC3 cells during AMF stimulation in the absence of extracellular Ca^{2+}. Images are displayed at 12 min intervals throughout the experiment. (b) Normalized fluorescence intensity (F/F_0) over time, showing the average intracellular Ca^{2+} levels during AMF stimulation.

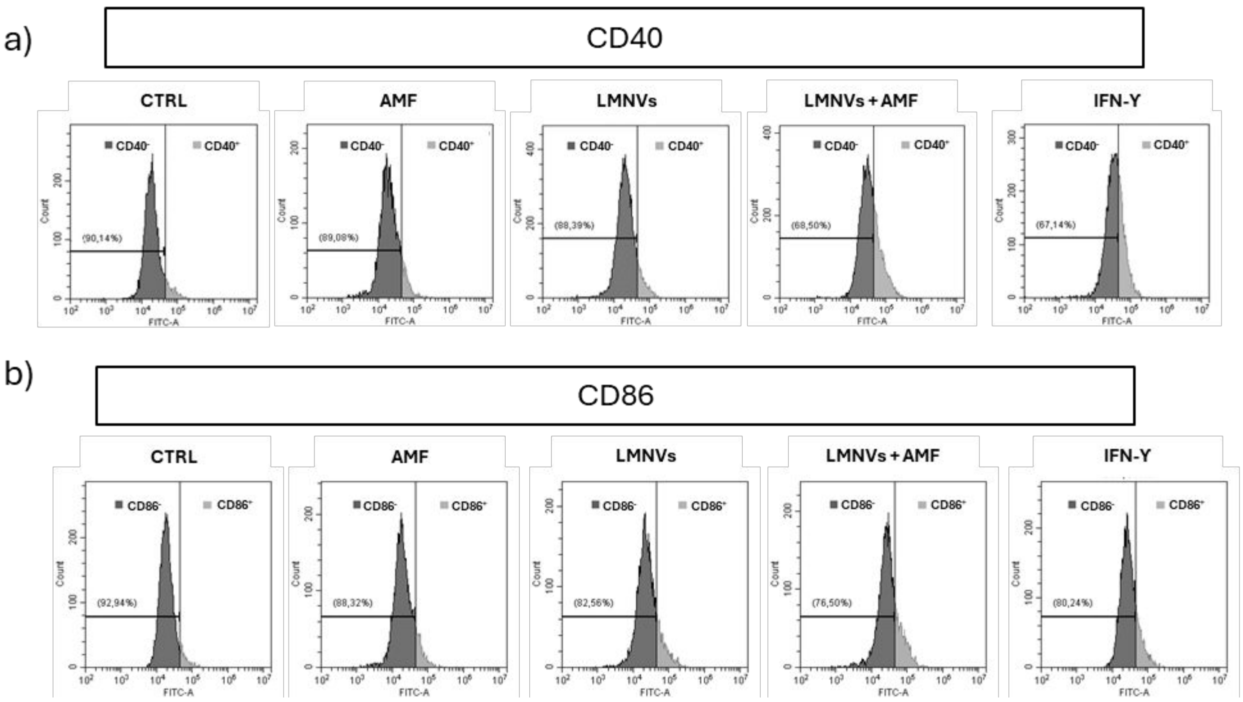

Figure S6. Representative flow cytometry plots of fluorescence levels indicative of the expression of (a) CD40 and (b) CD86 markers for each experimental condition.

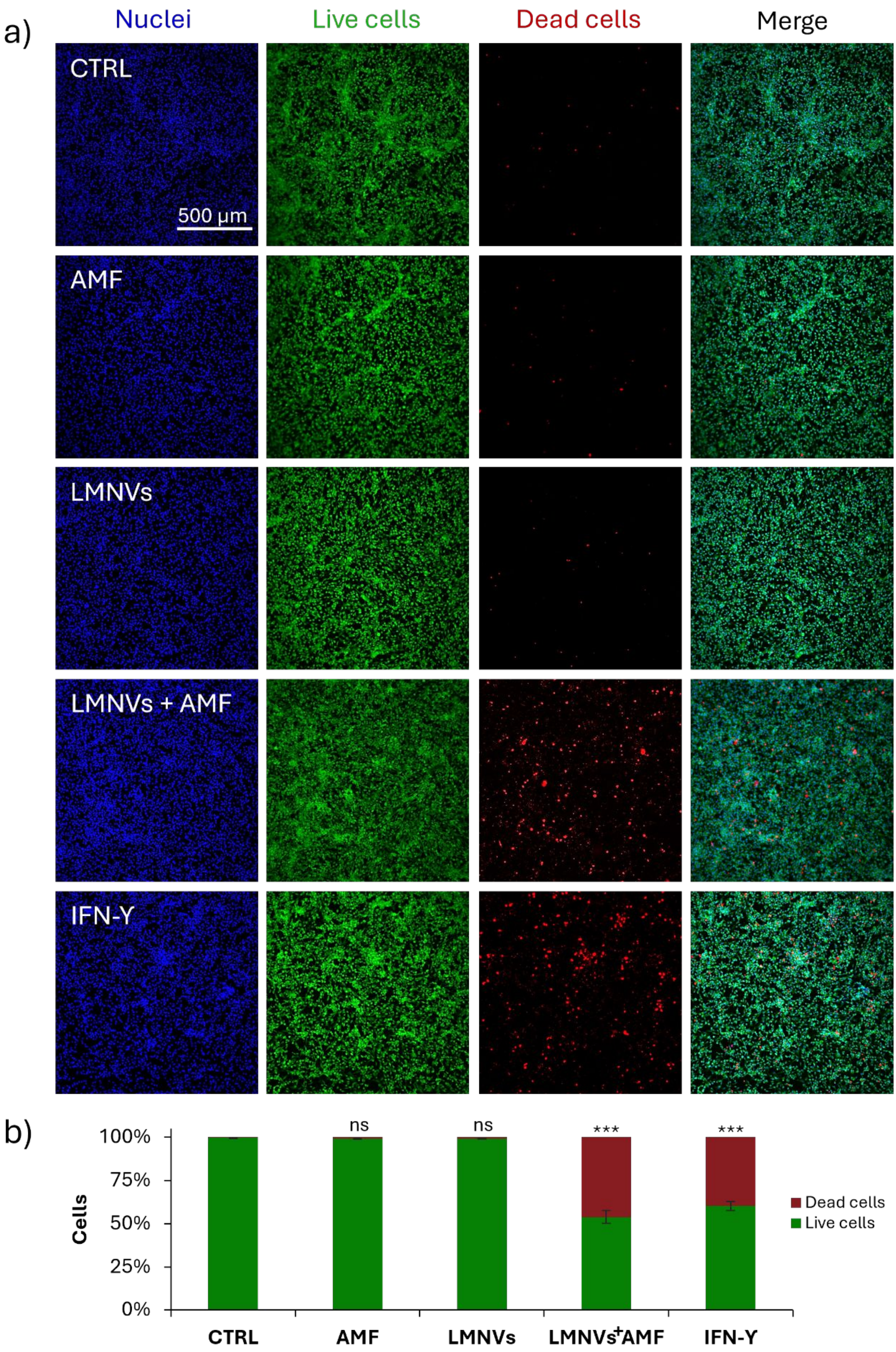

Figure S7. Cell viability in U87-MG cultures after microglia-conditioned medium treatment. (a) Representative confocal images and (b) quantitative analysis (ns $p > 0.05$, *** $p < 0.001$).

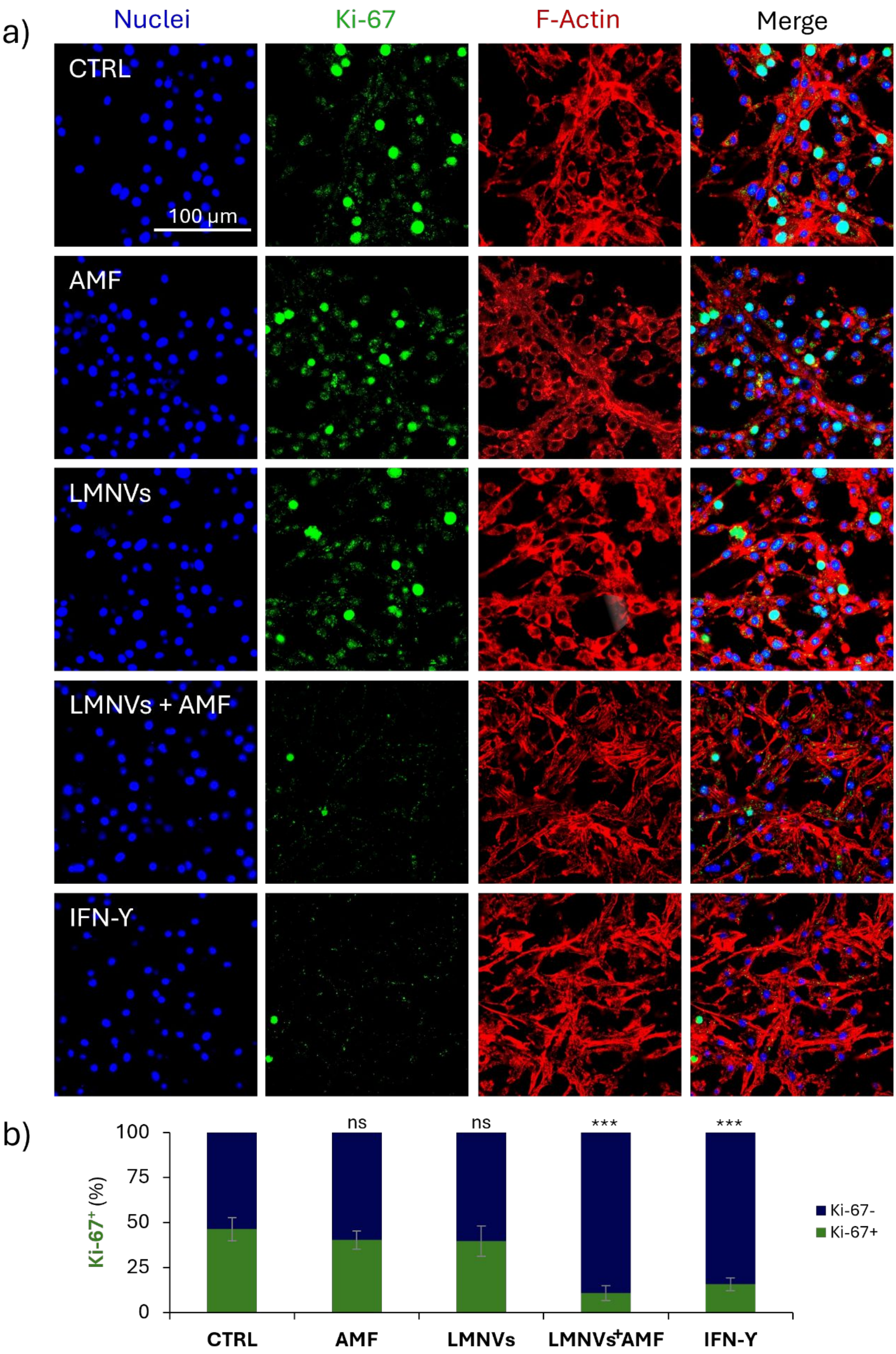

Figure S8. Cell proliferation activity in U87-MG cultures after microglia-conditioned medium treatment. (a) Representative confocal images and (b) quantitative analysis (ns $p > 0.05$, *** $p < 0.001$).

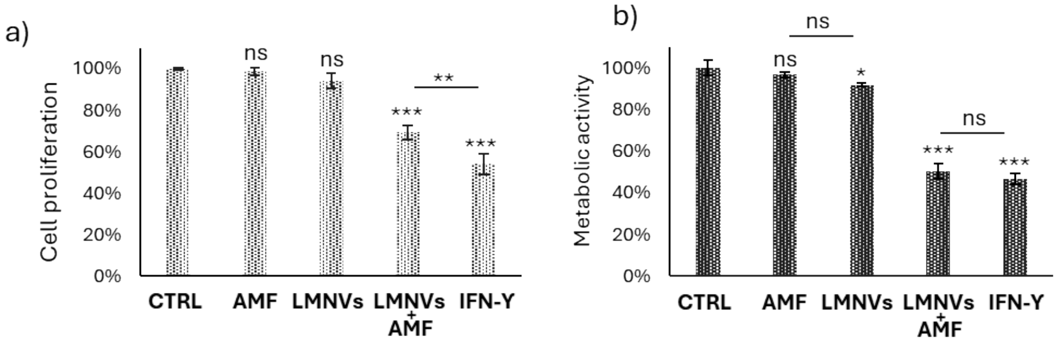

Figure S9. U87-MG cells exposed to microglia-conditioned media. Cell proliferation (a) and metabolic activity (b) for the different experimental groups (ns $p > 0.05$, * $p < 0.05$, ** $p < 0.01$, *** $p < 0.001$).

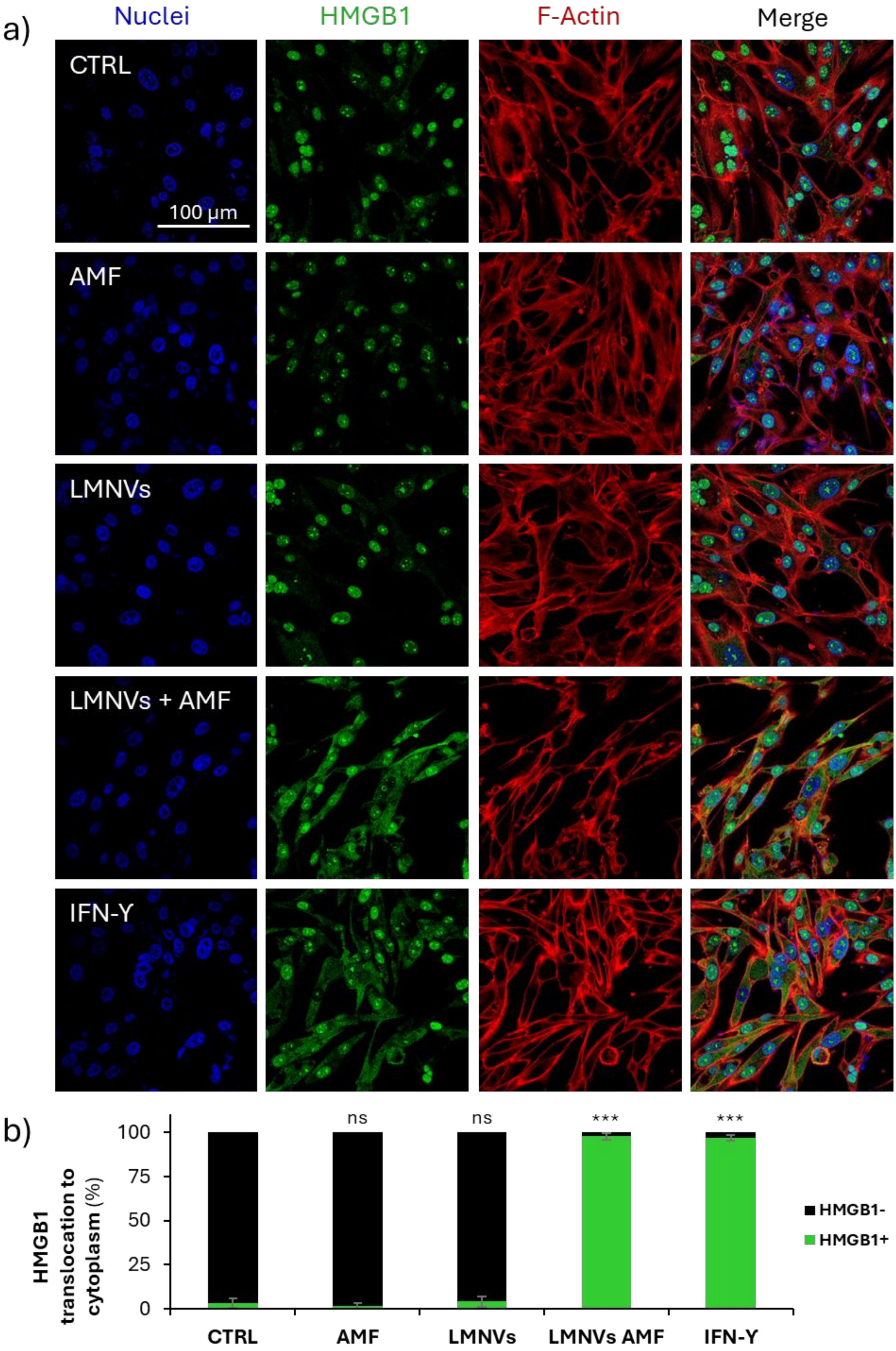

Figure S10. Immunostaining for HMGB1, a marker related to immunogenic cell death (ICD), after the treatment of U87-MG cells with microglia-conditioned medium. (a) Representative confocal images and (b) quantitative analysis considering the HMGB1 translocation from nuclei to cytoplasm (ns $p > 0.05$, *** p <0.001).

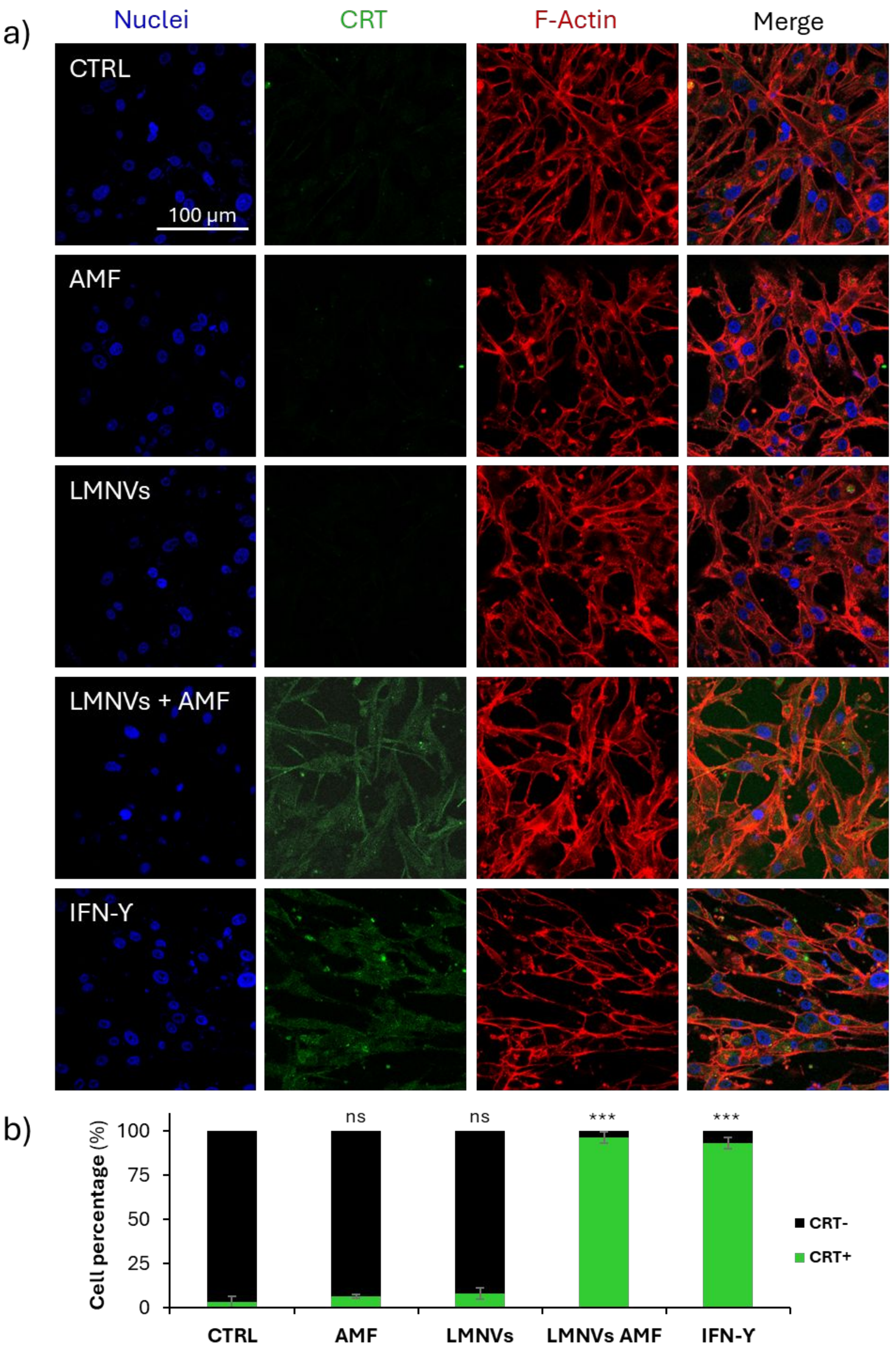

Figure S11. Immunostaining for calreticulin (CRT), a marker related to immunogenic cell death (ICD), after the treatment of U87-MG cells with microglia-conditioned medium. (a) Representative confocal images and (b) quantitative analysis (ns $p > 0.05$, *** $p < 0.001$).

Comparison	Upregulated genes	Downregulated genes	Total DEGs
AMF *vs.* CTRL	695	767	1462
LMNVs *vs.* CTRL	135	226	361
LMNVs + AMF *vs.* CTRL	279	365	644
IFN-γ *vs.* CTRL	622	490	1112

Table S1. Summary of the differentially expressed genes (DEGs) identified in each comparison, including the number of upregulated and downregulated genes.
